# Cross-coupling of dissimilar ketone enolates via enolonium species to afford non-symmetrical 1,4-diketones

**DOI:** 10.3762/bjoc.14.84

**Published:** 2018-05-03

**Authors:** Keshaba N Parida, Gulab K Pathe, Shimon Maksymenko, Alex M Szpilman

**Affiliations:** 1 Department of Chemical Sciences, Ariel University, 4070000 Ariel, Israel

**Keywords:** 1,4-diketones, enolates, enolonium species, hypervalent iodine, ketones, umpolung

## Abstract

Due to their closely matched reactivity, the coupling of two dissimilar ketone enolates to form a 1,4-diketone remains a challenge in organic synthesis. We herein report that umpolung of a ketone trimethylsilyl enol ether (1 equiv) to form a discrete enolonium species, followed by addition of as little as 1.2–1.4 equivalents of a second trimethylsilyl enol ether, provides an attractive solution to this problem. A wide array of enolates may be used to form the 1,4-diketone products in 38 to 74% yield. Due to the use of two TMS enol ethers as precursors, an optimization of the cross-coupling should include investigating the order of addition.

## Introduction

Substituted 1,4-dicarbonyl compounds are key intermediates for the preparation of numerous natural products and active pharmaceutical ingredients (APIs) with important biological activities. This is due to the facile conversion of 1,4-dicarbonyl compounds into five-membered heterocycles such as thiophenes, furans, and pyrroles. Consequently, numerous multistep approaches to unsymmetrical 1,4-dicarbonyl compounds involving, e.g., S_N_2-type displacements [1] or highly functionalized substrates such as β-ketoesters [2–3] or β-ketosulfones [4] have been developed. Recently, Loh reported the palladium-catalyzed coupling of an acid chloride with premade isolable indium homoenolates (In(CH_2_CHRC=OR')_2_), 1.2 equiv relative to the acid chloride) to give the corresponding 1,4-diketones [5]. Yet the direct coupling of two enolates is inarguably the shortest and most direct path to 1,4-dicarbonyl compounds. However, while oxidative dimerization of enolates is fairly straightforward [6–7], the coupling of two dissimilar enolates is contrastingly highly challenging. The more similar in steric and electronic properties the two dissimilar enolates become, the more difficult it becomes to achieve a selective cross-coupling rather than a statistical mixture of the two dimers and the desired unsymmetrical adduct. This may be overcome by using a large excess of one of the coupling partners [8–10], but this approach reduces the overall efficiency of the process. Thomson [11] and Wirth [6–7,12] both circumvented the selectivity and reactivity problem by making use of a temporary silicon connection strategy to render the reaction intramolecular. In both cases, the two ketone enolates were coupled successively to dimethyldichlorosilane. Thomson achieved the cross-coupling by using cerium(IV) as a one-electron oxidant [11]. Importantly for the discussion of the present work, Wirth’s strategy relied on a hypervalent iodine [13–15] mediated oxidative cross-coupling. Although these processes add a further step to the process, carrying out the cross-coupling in an intramolecular fashion has the double advantage of avoiding homocoupling as well as helping to overcome low reactivity in hindered systems such as cyclohexanone [12].

**Scheme 1:**
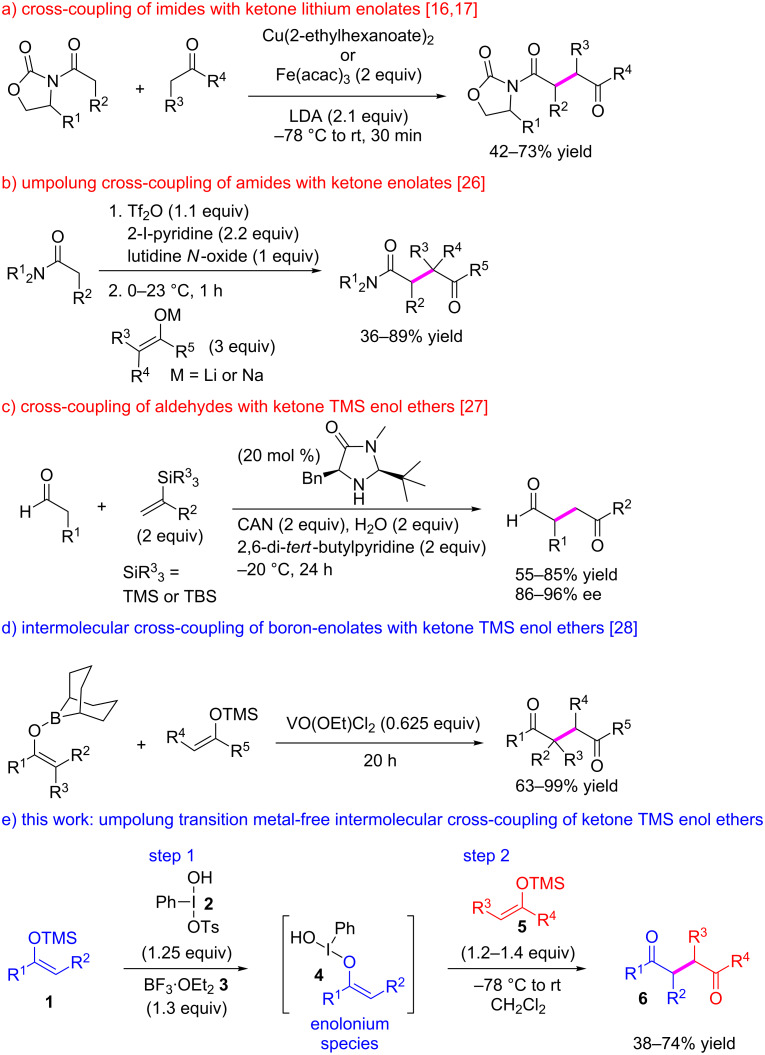
Oxidative intermolecular cross-coupling of dissimilar enolates.

To the best of our knowledge there are only five examples of successful intermolecular couplings of dissimilar enolates. Two of these examples involve the coupling of amide enolates with ketone enolates. Baran reported that stoichiometric Cu(2-ethylhexanoate)_2_ or Fe(acac)_3_ (2 equiv) are able to selectively oxidize imides, including Evan’s-type chiral imides, to the corresponding radicals. The formed radical then reacts selectively with a ketone lithium enolate followed by a second SET step to complete the transformation (Scheme 1a) [16–17]. A different approach, developed by Maulide, relies on the highly efficient umpolung of amides into enolonium species using triflic anhydride, a pyridine base and pyridine *N-*oxides (Scheme 1b). These enolonium species have been shown to react intramolecularly with *N*-benzyl groups [18–24]. The same principle has also been applied to the α-oxidation of amides [25]. Recently, Maulide showed that this powerful concept provides as solution to the coupling of amides with a large variety of ketone enolates to give 1,4-dicarbonyl compounds [26]. However, the radical-based method of Baran and the umpolung method of Maulide both take advantage of the selective activation of an amide and are therefore not amenable to ketone enolates. MacMillan reported the organocatalytic oxidative enantioselective coupling of in situ formed aldehyde enamines with excess (2 equiv) trialkylsilyl enol ethers (Scheme 1c) [27]. This reaction was proposed to proceed through a mechanism involving the attack of an enamine radical on the trialkylsilyl enol ether.

The last two examples concern the even more challenging cross-coupling of two dissimilar ketone enolates. In this context Hirao achieved the intermolecular cross-coupling by taking advantage of the different oxidation potentials of boron enolates and trimethylsilyl enol ethers to achieve selectivity with vanadium (V, 0.625 equiv) as the oxidant (Scheme 1d) [28–30]. The final example is the intermolecular cross-coupling of two dissimilar trimethylsilyl enol ethers described herein (Scheme 1e).

## Results and Discussion

**Scheme 2:**
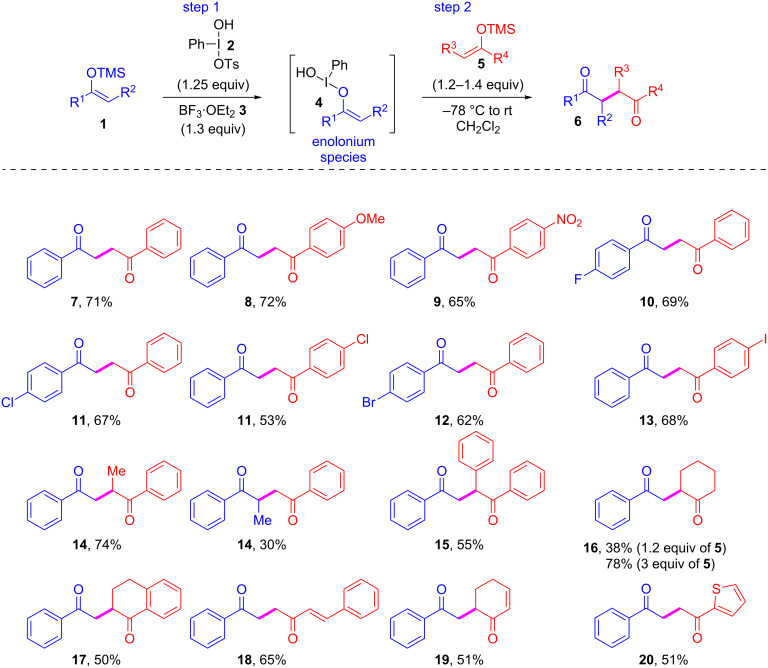
Scope of the homo- and heterocoupling of enolates. The purple bond indicates the bond formed. The blue-colored fragments indicate the first TMS enol ether used to produce the electrophilic enolonium species **4** and the red fragments indicate the second TMS enol ether used as nucleophile **5**. All yields are isolated yields.

Previously we have established that as little as 1.25 equiv of Koser’s reagent and 1.25 equiv of boron trifluoride in dichloromethane constituted an optimal recipe for preparing a variety of enolonium species. We have also shown that the enolonium species **4** (R^1^ = Ph, R^2^ = H) can be produced from the corresponding TMS enol ether **1** (R^1^ = Ph, R^2^ = H) and subsequently coupled with a second molecule of enol ether **1/5** (R^1/3^ = Ph, R^2/4^ = H) to afford the 1,4-diketone **7** in 71% yield (Scheme 2) [31]. We therefore focused on identifying the minimum amount of the second enolate that would lead to optimal yields and found that as little as 1.2–1.4 equiv provided the desired 1,4-diketones in acceptable yields without the need for a large excess of the second coupling partner (Scheme 2). Since the two coupling partners are both trimethylsilyl enol ethers, an advantage of this method is that the optimization of the coupling of a given enolate pair may be investigated by simply reversing the order of addition. In general, the major competing side reaction was the nucleophilic attack by the tosylate on the enolonium species **4**. Only in the rare cases mentioned below homocoupling did take place.

The enolonium species **4** (R^1^ = Ph, R^2^ = H) reacts readily with both electron-rich and electron-poor TMS enol ethers **5** (Scheme 2). Thus, the cross-coupling of **4** (R^1^ = Ph, R^2^ = H) with the TMS enol ether **5** (R^3^ = *p*-MeOC_6_H_4_, R^4^ = H) afforded compound **8** in 72% yield with no oxidation of the electron-rich aromatic ring observed. The only side product being tosyloxy-acetophenone. The same enolonium species **4** (R^1^ = Ph, R^2^ = H) reacts with the TMS enol ether **5** (R^3^ = *p*-O_2_NC_6_H_4_, R^4^ = H) to form **9** in 65% yield.

The method is by no means restricted to enolonium species of acetophenone as may be observed from the formation of the whole series of *para-*halogenated 1,4-diketones. Thus, the *p-*fluoro-, *p-*chloro-, and *p-*bromo-substituted enolonium species **4** (R^3^ = *p-*X-C_6_H_4_, R^4^ = H, X = F, Cl, or Br) were generated and used in the cross-coupling with TMS enol ether **5** (R^3^ = Ph, R^4^ = H) to give the products in 69% (**10**), 67% (**11**), and 62% (**12**) yield, respectively. In case of the reverse addition, namely reacting enolonium species **4** (R^1^ = Ph, R^2^ = H) with TMS enol ether **5** (R^3^ = *p-*Cl-C_6_H_5_, R^4^ = H), product **11** was isolated in 53% yield. Therefore, for non-commercially available enolates it could be advantageous to try both orders of addition to achieve an optimal result. Indeed, when the iodo-substituted enolonium species **4** (R^1^ = *p-*I-C_6_H_4_, R^2^ = H) was prepared and reacted with TMS enol ether **5** (R^3^ = Ph, R^4^ = H) the desired product **13** is obtained in less than 10% yield accompanied with the homo-dimers and tosyloxylated ketones as byproducts. In contrast, reversing the order of addition of the TMS-enol ethers afforded **13** in 68% yield. It should be noted that we have previously used the enolonium species **4** (R^1^ = *p-*I-C_6_H_4_, R^2^ = H) successfully in the coupling with 2-methylindole and *N*-methyl-2-methylindole and obtained the products in 77% and 74% yield, respectively [32]. The low yield achieved here therefore reflects issues unique to the reaction with the second enolate and not the stability of the enolonium species itself. Based on the formation of dimers of both ketones used, we speculate that this is due to partial oxidation of the second enolate by **4** (R^1^ = *p-*I-C_6_H_4_, R^2^ = H), perhaps due to relatively slower cross-coupling. Irrespective, these issues are easily avoided simply by using the reverse addition to give the desired product **13** in good yield.

The choice of the order of addition is also of importance for substrates with more sterical hindrance. Here the trend is clear: it is advantageous to use the less sterically hindered TMS enol ether to generate the enolonium species **4** followed by the addition of the more sterically hindered TMS enol ether **5**. For example, the addition of enolonium species **4** (R^1^ = Ph, R^2^ = Me) to the TMS enol ether **5** (R^3^ = Ph, R^4^ = H) led to formation of the product **14** in 30% yield with significant formation of 1-tosyloxypropiophenone as the major byproduct. However, when the order of addition was reversed, i.e., the enolonium species **4** (R^1^ = Ph, R^2^ = H) was cross-coupled with TMS enol ether **5** (R^3^ = Ph, R^4^ = Me) the same product **14** was obtained in 74% yield. Apparently, in these cases the sterically hindered nature of the enolonium species leads it to react faster with the less-hindered tosylate despite its poor electronic nucleophilicity. Thus, when the strategy of converting the least hindered enolate into the enolonium species **4** is used even highly hindered TMS enol ethers **5** may be used with formation of tertiary carbon centers. Thus, the enolonium species **4** (R^1^ = Ph, R^2^ = H) reacted with TMS enol ether **5** (R^1^ = Ph, R^2^ =Ph) to afford the cross-coupling product **15** in 55% yield. The same enolonium species could be cross-coupled with the TMS enol ether of cyclohexanone to afford the product **16** in 38% yield. Reversing the order of addition in this case led to only trace amounts of the desired cross-coupling product. On the other hand, using 3 equiv of cyclohexanone TMS enol ether, similarly to the conditions used by Maulide (Scheme 1b) [26] and MacMillan (Scheme 1c) [27], led to the product **16** in 78% yield. The cross-coupling of **4** (R^1^ = Ph, R^2^ = H) with the hindered TMS enol ether of tetralone (1.2 equiv) afforded **17** in 50% yield.

Importantly, also double-bond containing ketones may be used in the reaction. For example, **4** (R^1^ = Ph, R^2^ = H) reacted with the TMS enol ether of cyclohexenone to give **19** in 51% yield and the reaction with the TMS enol ether of (*E*)-4-phenylbut-3-en-2-one led to formation and isolation of **18** in 65% yield (Scheme 2). Given the ubiquity of heterocycles in natural products and modern APIs it is also of importance that cross-coupling of the easily oxidized TMS enol ether **5** (R^3^ = 2-thiophenyl, R^4^ = H) with **4** (R^1^ = Ph, R^2^ =H) afforded **20** in 51% yield.

**Scheme 3:**
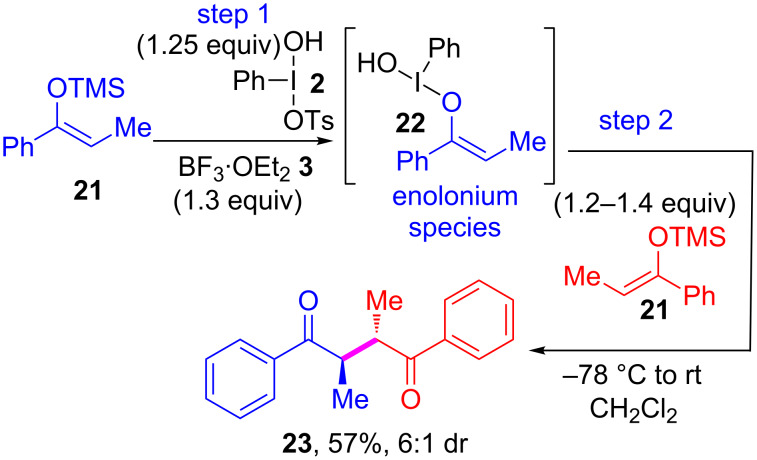
Study of diastereoselectivity of the cross-coupling reaction.

Since both *meso***-23** and *rac*-**23** are well-described in the literature [33], we chose to study the diastereoselectivity of the reaction using the dimerization of enol ether **21**. The geometry of the TMS enol ether **21** was established as being exclusively *Z* based on 2D-NOE NMR (Scheme 3). The enolate **21** was then converted into enolonium species **22** and cross-coupled with a second equivalent of **21** to give the two separable diastereoisomers of **23** one *meso* and one *rac* in 6:1 diastereoselectivity*.* This in conjunction with earlier work from our group [34] indicates that enolonium species of type **4** (Scheme 1 and Scheme 2) are mostly configurationally stable under the conditions used.

## Conclusion

We have shown that a two-step strategy, involving the formation of the enolonium species in the first step and attack by a nucleophilic TMS enol ether in the second step provides a powerful method for intermolecular cross-coupling of dissimilar trimethylsilyl enol ethers. Only 1.2–1.4 equiv of the second enolate is needed. Despite the low ratio between the two reacting dissimilar enolates used, the products are formed in good yield in a single operation and with good diastereoselectivity. We hope that the ease of carrying out and optimizing the procedure will make it useful for chemists interested in making unsymmetrical 1,4-diketones.

## Supporting Information

File 1 Experimental, characterization data and copies of NMR spectra.
