# Supplementary material for: Cross-coupling of dissimilar ketone enolates via enolonium species to afford non-symmetrical 1,4-diketones
Source: Beilstein J Org Chem. 2018 May 3;14:992–7. doi: 10.3762/bjoc.14.84 (PMC6009205; doi:10.3762/bjoc.14.84)
Supplement: File 1 — Experimental, characterization data and copies of NMR spectra. [file Beilstein_J_Org_Chem-14-992-s001.pdf]

Supporting Information

for

Cross-coupling of dissimilar ketone enolates via enolonium species to afford non-symmetrical 1,4-diketones

Keshaba N. Parida, Gulab K. Pathe, Shimon Maksymenko, and Alex M. Szpilman*

Address: Department of Chemical Sciences, Ariel University, 4070000 Ariel, Israel

Email: Alex M. Szpilman - amszpilman@gmail.com

*Corresponding author

Experimental, characterization data and copies of NMR spectra

Table of contents:

General methods S2

General procedure for the synthesis of 1,4-diketones.. S2

Experimental procedures and characterization data S3

References.. S9

NMR spectra S10

General methods: Air and/or moisture-sensitive reactions were carried out in anhydrous solvents under an argon atmosphere in predried glassware. CH_2Cl_2 was freshly distilled from CaH_2. All commercially available reagents were used without purification. Column chromatography was carried out on silica gel (100–200 mesh). NMR spectra were recorded on a Bruker Avance III 400 spectrometer operating at 400 MHz (^{1}H) and 101 MHz (^{13}C). ^{1}H and ^{13}C NMR chemical shifts are reported in ppm relative to chloroform-D (δ = 7.26 for ^{1}H and 77.16 for ^{13}C) or TMS (0.0 ppm) and coupling constants (J) are reported in hertz (Hz). The following abbreviations have been used to designate signal multiplicity: s = singlet, d = doublet, t = triplet, q = quartet, m = multiplet, br = broad. IR spectra were recorded as films on a Bruker FT-IR. TOF-ESI-HSMS (*m*/*z*) was recorded on a Waters Micromass LCT premier instrument at 70 eV in the positive or negative mode. APCI-HRMS was recorded on a Bruker Maxis Impact QTOF instrument using the APCI solid probe.

General procedure for the synthesis of 1,4-diketones

Under an argon atmosphere, $BF_3 \cdot OEt_2$ (1.25 equiv) was added to a slurry of hydroxy(tosyloxy)iodobenzene (1.25 equiv) in anhydrous CH_2Cl_2 (8 mL) at room temperature resulting in a clear yellow solution. The solution was cooled to −78 °C, and the first trimethylsilyl enol ether (1.0 equiv) dissolved in CH_2Cl_2 (4 mL) was added slowly. After 10 minutes the second trimethylsilyl enol ether (1.2–1.4 equiv) dissolved in CH_2Cl_2 (2 mL) was added dropwise. The reaction mixture was stirred for 10 min at −78 °C and then allowed to warm to room temperature. Stirring was continued for 1 h in total. The reaction mixture was quenched by the addition of H_2O (5 mL) and then extracted with CH_2Cl_2 (3 × 10 mL). The combined organic extracts were dried over anhydrous Na_2SO_4 and concentrated in vacuo. The residue was subjected to silica gel column chromatography to isolate the corresponding 1,4-diketone product(s).

1-(4-Methoxyphenyl)-4-phenylbutane-1,4-dione (8): Cross-coupling of 1-phenyl-1-trimethylsiloxyethylene (192 mg, 1.0 mmol) with 1-(4-methoxyphenyl)-1-trimethylsiloxyethylene (311 mg, 1.4 mmol) following the general procedure gave 1-(4-methoxyphenyl)-4-phenylbutane-1,4-dione (193 mg, 72%) as white solid; **R*f***: 0.4 (1:4 v/v EtOAc/hexane). **FT-IR**: V*max* 2979, 1710, 1678, 1599, 1356, 1171, 954 cm^{-1}. **^{1}H NMR** (400 MHz, $CDCl_3$) δ 7.97–8.08 (m, 4H), 7.54–7.61 (m, 1H), 7.45–7.52 (m, 2H), 6.92–6.98 (m, 2H), 3.88 (s, 3H), 3.37–3.51 (m, 4H) ppm. **^{13}C NMR** (101 MHz, $CDCl_3$) δ 199.1, 197.4, 163.7, 137.0, 133.3, 130.5, 130.1, 128.7, 128.3, 113.9, 55.6, 32.8, 32.4 ppm; **HRMS** ($APCI^+$): *m/z* calculated for $C_{17}H_{17}O_3$ 269.1178; $[M+H]^+$ found 269.1192.

1-(4-Nitrophenyl)-4-phenylbutane-1,4-dione (9): Cross-coupling of 1-phenyl-1-trimethylsiloxyethylene (192 mg, 1.0 mmol) with 1-(4-nitrophenyl)-1-trimethylsiloxyethylene (332 mg, 1.4 mmol) following the general procedure gave 1-(4-nitrophenyl)-4-phenylbutane-1,4-dione (184 mg, 65%) as an yellow solid; **R*f***: 0.4 (1:4 v/v EtOAc/hexane). **FT-IR**: V*max* 2968, 1662, 1303, 959, 864 cm^{-}; **^{1}H NMR** (400 MHz, $CDCl_3$): δ 8.28–8.37 (m, 2H), 8.15–8.24 (m, 2H), 7.97–8.09 (m, 2H), 7.56–7.65 (m, 1H), 7.44–7.54 (m, 2H), 3.41–3.56 (m, 4H) ppm. **^{13}C NMR** (101 MHz, $CDCl_3$): δ 198.3, 197.5, 150.5, 141.5, 136.6, 133.6, 129.3, 128.8, 128.3, 124.0, 33.2, 32.8 ppm; **HRMS** ($APCI^+$): *m/z* calculated for $C_{16}H_{14}N_4O$ 284.0923; $[M+H]^+$ found 284.0949.

1-(4-Fluorophenyl)-4-phenylbutane-1,4-dione (10) [1]: Cross-coupling of 1-(4-fluorophenyl)-1-trimethylsiloxyethylene (262 mg, 1.246 mmol) with 1-phenyl-1-trimethylsiloxyethylene (336 mg, 1.747 mmol) following the general procedure gave 1-(4-fluorophenyl)-4-phenylbutane-1,4-dione (219 mg, 69%) as colorless crystal. **R*f***: 0.4 (1:9 v/v EtOAc/hexane). **^{1}H NMR** (400 MHz, $CDCl_3$): δ 8.02–8.09 (m, 4H), 7.58 (tt, *J* = 7.28, 1.2 Hz, 1H), 7.48 (tt, *J* = 7.28, 1.64 Hz, 2H), 7.15 (tt, *J* = 8.62, 2.1 Hz, 2H), 3.42–3.47 (m, 4H) ppm.

^{13}C NMR (101 MHz, $CDCl_3$): 198.7, 197.2, 166.0 (d, *J* = 254.6 Hz), 136.9, 133.4, 130.5 (d, *J* = 9.3 Hz), 128.8, 128.7, 128.3, 115.8 (d, *J* = 21.9 Hz), 32.7, 32.6 ppm.

1-(4-Chlorophenyl)-4-phenylbutane-1,4-dione (11)[2]: Cross-coupling of 1-(4-chlorophenyl)-1-trimethylsiloxyethylene (283 mg, 1.248 mmol) with 1-phenyl-1-trimethylsiloxyethylene (336 mg, 1.747 mmol) following the above general procedure gave 1-(4-chlorophenyl)-4-phenylbutane-1,4-dione (229 mg, 67%) as a colorless solid. **R_f**: 0.4 (1:9 v/v EtOAc/hexane). **^{1}H NMR** (400 MHz, $CDCl_3$): δ 8.02–8.05 (m, 2H), 7.96–8.00 (m, 2H), 7.58 (tt, *J* = 7.4, 1.3 Hz, 1H), 7.44–7.50 (m, 4H), 3.40–3.49 (m, 4H) ppm. **^{13}C NMR** (101 MHz, $CDCl_3$): δ 198.6, 197.6, 139.7, 136.8, 135.3, 133.4, 129.7, 129.1, 128.8, 128.3, 32.7, 32.4 ppm.

1-(4-Bromophenyl)-4-phenylbutane-1,4-dione (12): Cross-coupling of 1-(4-bromophenyl)-1-trimethylsiloxyethylene (337 mg, 1.242 mmol) with 1-phenyl-1-trimethylsiloxyethylene (336 mg, 1.747 mmol) following the above general procedure gave 1-(4-bromophenyl)-4-phenylbutane-1,4-dione (246 mg, 62%) as a colorless solid. **R_f**: 0.4 (1:9 v/v EtOAc/hexane). **FT-IR**: V_{max} 2905, 1673, 1583, 1319, 991, 742, 689 cm^{-1}. **^{1}H NMR** (400 MHz, $CDCl_3$): δ 8.02–8.05 (m,2H), 7.88–7.92 (m, 2H), 7.63 (td, *J* = 8.64, 1.96 Hz, 2H), 7.58 (tt, *J* = 7.4, 1.96 Hz, 1H), 7.48 (tt, *J* = 7.32, 1.64 Hz, 2H), 3.42–3.47 (m, 4H) ppm. **^{13}C NMR**: (101 MHz, $CDCl_3$): δ 198.6, 197.8, 136.8, 135.7, 133.4, 132.1, 129.82, 129.8, 128.5, 128.3, 32.7, 32.6 ppm. **HRMS (ESI+)**: *m*/*z* calculated for $C_{16}H_{14}BrO_2$ 317.0177 $[M+H]^+$; found 317.0198.

1-(4-Iodophenyl)-4-phenylbutane-1,4-dionene (13): Cross-coupling of 1-phenyl-1-trimethylsiloxyethylene (192 mg, 1.0 mmol) with 1-(4-iodophenyl)-1-trimethylsiloxyethylene (440 mg, 1.382 mmol) following the general procedure gave 1-(4-iodophenyl)-4-phenylbutane-1,4-dionene (248 mg, 68%) as an white solid; R_f: 0.5 (1:5 v/v EtOAc/hexane). **FT-IR**: V_{max} 2921, 1705, 1678, 1599, 1451, 1350, 922 cm^{-1}. **^{1}H NMR** (400 MHz, $CDCl_3$) *δ*

7.98–8.09 (m, 2H), 7.79–7.91 (m, 2H), 7.69–7.79 (m, 2H), 7.52–7.64 (m, 1H), 7.42–7.52 (m, 2H), 3.36–3.51 (m, 4H) ppm. **^{13}C NMR** (101 MHz, $CDCl_3$) δ 198.6, 198.2, 138.1, 136.8, 136.2, 133.4, 129.7, 129.66, 128.8, 128.3, 101.2, 32.7, 32.6 ppm; **HRMS** ($APCI^+$) *m*/*z* calculated for $C_{16}H_{14}IO_2$ 365.0039; $[M+H]^+$ found 365.0061.

2-Methyl-1,4-diphenylbutane-1,4-dione (14):[2] Cross-coupling of 1-phenyl-1-trimethylsiloxyethylene (240 mg, 1.248 mmol) with trimethyl((1-phenylprop-1-en-1-yl)oxy)silane (360 mg, 1.747 mmol) following the above general procedure gave 2-methyl-1,4-diphenylbutane-1,4-dione (233 mg, 74%) as a colorless solid; **R_f**: 0.4 (1:9 v/v EtOAc/hexane). **^{1}H NMR** (400 MHz, $CDCl_3$): δ 8.06 (dt, *J* = 8.5, 1.7 Hz, 2H), 7.99 (dt, *J* = 8.5, 1.7 Hz, 2H), 7.54–7.60 (m, 2H), 7.43–7.51 (m, 4H), 4.19 (ddd, *J* = 8.4, 7.2, 4.9 Hz, 1H), 3.74 (dd, *J* = 18.0, 8.5 Hz, 1H), 3.12 (dd, *J* = 18.0, 4.9 Hz, 1H), 1.29 (d, *J* = 7.2 Hz, 3H) ppm. **^{13}C NMR** (101 MHz, $CDCl_3$): δ 203.5, 198.6, 136.7, 136.2, 133.3, 133.1, 128.8, 128.7, 128.6, 128.2, 42.5, 36.4, 18.1 ppm.

1,2,4-Triphenylbutane-1,4-dione (15)[3]: Cross-coupling of 1-phenyl-1-trimethylsiloxyethylene (192 mg, 1.00 mmol) with ((1,2-diphenylvinyl)oxy)trimethylsilane (376 mg, 1.400 mmol) following the general procedure gave 1,2,4-triphenylbutane-1,4-dione (173 mg, 55%) as an colorless oil. **R_f**: 0.5 (1:5 v/v EtOAc/hexane). **^{1}H NMR** (400 MHz, $CDCl_3$): δ 7.95–8.08 (m, 4H), 7.28–7.60 (m, 10H), 7.18–7.26 (m, 1H), 5.34 (dd, *J* = 10.0, 3.7 Hz, 1H), 4.23 (dd, *J* = 18.0, 10.1 Hz, 1H), 3.31 (dd, *J* = 18.0, 3.7 Hz, 1H) ppm. **^{13}C NMR** (101 MHz, $CDCl_3$): δ 199.1, 198.2, 138.8, 136.6, 133.4, 133.0, 129.3, 129.1, 128.8, 128.6, 128.59, 128.4, 128.3, 127.5, 48.9, 44.0 ppm.

2-(2-Oxo-2-phenylethyl)cyclohexan-1-one (16)[4]: Cross-coupling of 1-phenyl-1-trimethylsiloxyethylene (240 mg, 1.248 mmol) with trimethylsilyl enolether of cyclohexanone (255 mg, 1.49 mmol) following the above general procedure gave 2-(2-oxo-2-

phenylethyl)cyclohexan-1-one (103 mg, 38%) as colorless solid. When 3 equivalents (637 mg, 3.744 mmol) of trimethylsilyl enol ether of cyclohexanone was used the yield was 78% (211 mg). **R*f***: 0.24 (1:4 v/v Et_2O/Pentane). **^{1}H NMR** (400 MHz, $CDCl_3$): δ 7.98 (d, 2H), 7.55 (t, 1H), 7.45 (t, 2H), 3.60 (dd, *J* = 17.7, 6.6 Hz, 1H), 3.10–3.23 (m, 1H), 2.68 (dd, *J* = 17.7, 5.7 Hz, 1H), 2.43 (dd, *J* = 9.8, 5.0 Hz, 2H), 2.06–2.24 (m, 2H), 1.56–1.94 (m, 3H), 1.45 (qd, *J* = 12.8, 3.8 Hz, 1H) ppm. **^{13}C NMR** (101 MHz, $CDCl_3$): δ 211.7, 198.8, 137.2, 133.13, 128.6, 128.2, 46.6, 42.1, 38.4, 34.4, 28.1, 25.5 ppm.

2-(2-Oxo-2-phenylethyl)-3,4-dihydronaphthalen-1(2*H*)-one (17)[5]: Cross-coupling of 1-phenyl-1-trimethylsiloxyethylene (192 mg, 1.00 mmol) with ((3,4-dihydronaphthalen-1-yl)oxy)trimethylsilane (306 mg, 1.400 mmol) following the general procedure gave 2-(2-oxo-2-phenylethyl)-3,4-dihydronaphthalen-1(2*H*)-one (133 mg, 50%) as an white solid; **R*f***: 0.6 (1:5 v/v EtOAc/hexane); **^{1}H NMR** (400 MHz, $CDCl_3$): *δ* 7.97–8.10 (m, 3H), 7.52–7.65 (m, 1H), 7.42–7.52 (m, 3H), 7.24–7.34 (m, 2H), 3.87 (dd, *J* = 17.5, 4.6 Hz, 1H), 3.33 (ddt, *J* = 13.3, 7.1, 4.6 Hz, 1H), 3.19 (ddd, *J* = 17.0, 12.7, 4.5 Hz, 1H), 2.99 (ddd, *J* = 17.6, 7.2, 2.9 Hz, 2H), 2.25–2.36 (m, 1H), 1.99 (qd, *J* = 13.0, 4.3 Hz, 1H) ppm. **^{13}C NMR** (101 MHz, $CDCl_3$): *δ* 199.2, 198.7, 144.3, 137.2, 133.5, 133.3, 132.5, 128.9, 128.8, 128.3, 127.7, 126.8, 44.4, 39.2, 29.7, 29.6 ppm.

1,6-Diphenylhex-5-ene-1,4-dione (18): Cross-coupling of 1-phenyl-1-trimethylsiloxyethylene (231 mg, 1.2 mmol) with trimethyl((4-phenylbuta-1,3-dien-2-yl)oxy)silane (366 mg, 1.678 mmol) following the above general procedure gave 1,6-diphenylhex-5-ene-1,4-dione (206 mg, 65%) as colorless solid. **R*f***: 0.26 (1:4 v/v Et_2O/pentane). **FT-IR**: 3041, 1686, 1619, 1395, 1353, 1207, 1103, 749 cm^{-}. **^{1}H NMR** (400 MHz, $CDCl_3$): δ 7.94–8.03 (m, 2H), 7.65 (d, *J* = 16.2 Hz, 1H), 7.57 (dd, *J* = 6.6, 2.9 Hz, 3H), 7.48 (t, *J* = 7.6 Hz, 2H), 7.37–7.43 (m, 3H), 6.83 (d, *J* = 16.2 Hz, 1H), 3.41 (t, *J* = 6.5 Hz,

2H), 3.17 (t, J = 6.5 Hz, 2H). **^{13}C NMR**: (101 MHz, $CDCl_3$): δ 198.8, 198.78, 143.0, 136.9, 134.7, 133.3, 130.6, 129.1, 128.7, 128.5, 128.3, 126.3, 34.6, 32.7 ppm. **HRMS (APCI+)**: *m/z* calculated for $C_{18}H_{17}O_2$ 265.1229 $[M+H]^+$; found 297.1517.

6-(2-Oxo-2-phenylethyl)cyclohex-2-enone (19): Cross-coupling of 1-phenyl-1-trimethylsiloxyethylene (240 mg, 1.248 mmol) with (cyclohexa-1,5-dien-1-yloxy)trimethylsilane (294 mg, 1.747 mmol) following the above general procedure gave 6-(2-oxo-2-phenylethyl)cyclohex-2-enone (136 mg, 51%) as a colorless liquid; **R_f**: 0.4 (1:3 v/v EtOAc/hexane). **FT-IR**: 2931, 2859, 1686, 1452, 1181, 1103, 983, cm^{-1}. **^{1}H NMR** (400 MHz, $CDCl_3$): δ 7.99 (dt, J = 8.5, 1.7 Hz, 2H), 7.54 (tt, J = 7.4, 1.3 Hz, 1H), 7.45 (tt, J = 7.3, 1.6 Hz, 2H), 6.97 (dddd, J = 9.7, 5.6, 2.3, 1.5 Hz, 1H), 5.05 (ddd, J = 10.0, 2.9, 1.0 Hz, 1H), 3.75 (dd, J = 17.6, 4.4 Hz, 1H), 3.12 (ddt, J = 13.5, 7.6, 4.5 Hz, 1H), 2.82 (dd, J = 17.6, 7.5 Hz, 1H), 2.35–2.60 (m, 2H), 2.15–2.20 (m, 1H), 1.82 (tdd, J = 13.3, 11.5, 5.1 Hz, 1H) ppm. **^{13}C NMR** (101 MHz, $CDCl_3$): δ 200.3, 198.7, 150.2, 137.1, 133.2, 129.5, 128.7, 128.2, 43.1, 38.6, 29.2, 26.2 ppm. **HRMS (APCI+)**: *m/z* calculated for $C_{14}H_{15}O_2$ 215.1072 $[M+H]^+$; found 215.1066.

1-Phenyl-4-(thiophen-2-yl)butane-1,4-dione (20)[6]: Cross-coupling of 1-phenyl-1-trimethylsiloxyethylene (240 mg, 1.248 mmol) with trimethyl((1-(thiophen-2-yl)vinyl)oxy)silane (346 mg, 1.747 mmol) following the above general procedure gave 1-phenyl-4-(thiophen-2-yl)butane-1,4-dione (156 mg, 51%) as a colorless solid; **R_f**: 0.4 (1:3 v/v EtOAc/hexane). **FT-IR**: 2926, 2869, 1681, 1655, 1400, 1348, 1181 cm^{-1}. **^{1}H NMR** (400 MHz, $CDCl_3$): δ 8.03 (dt, J = 7.1, 1.0 Hz, 2H), 7.84 (dd, J = 3.8, 1.1 Hz, 1H), 7.65 (dd, J = 5.0, 1.1 Hz, 1H), 7.58 (tt, J = 7.4, 1.2 Hz, 1H), 7.48 (tt, J = 7.3, 1.6 Hz, 2H), 7.16 (dd, J = 4.9, 3.8 Hz, 1H), 3.38–3.48 (m, 4H) ppm. **^{13}C NMR**: (101 MHz, $CDCl_3$): δ 198.6, 191.8, 144.1, 136.8, 133.7, 133.4, 132.2, 128.8, 128.3, 33.3, 32.4 ppm.

2,3-Dimethyl-1,4-diphenylbutane-1,4-dione (*meso*-23 and *rac*-23): Cross-coupling of trimethyl((1-phenylprop-1-en-1-yl)oxy)silane (257 mg, 1.247 mmol) with trimethyl((1-phenylprop-1-en-1-yl)oxy)silane (360 mg, 1.746 mmol) following the above general procedure gave 2,3-dimethyl-1,4-diphenylbutane-1,4-dione (188 mg, 57%), where *rac*:*meso* = 1:6.2.

***meso*-2,3-Dimethyl-1,4-diphenylbutane-1,4-dione (*meso*-23)**[7]: Colourless solid. **R_f**: 0.5 (1:9 v/v EtOAc/hexane). **^{1}H NMR** (400 MHz, $CDCl_3$): δ 8.05 (dt, *J* = 7.1, 1.4 Hz, 4H), 7.59 (tt, *J* = 7.4, 1.3 Hz, 2H), 7.50 (tt, *J* = 7.3, 1.6, 4H), 4.02–4.08 (m, 2H), 1.14 (dd, *J* = 4.5, 2.0 Hz, 6H) ppm. **^{13}C NMR**: (101 MHz, $CDCl_3$): δ 203.9, 137.0, 133.5, 128.9, 128.6, 43.5, 17.6 ppm.

***rac*-2,3-Dimethyl-1,4-diphenylbutane-1,4-dione (*rac*-23)**[7]: Colourless solid. **R_f**: 0.45 (1:9 v/v EtOAc/hexane). **^{1}H NMR** (400 MHz, $CDCl_3$): δ 7.99 (dt, *J* = 7.1, 1.4 Hz, 4H), 7.55 (tt, *J* = 7.4, 1.3 Hz, 2H), 7.46 (tt, *J* = 7.3, 1.6, 4H), 3.93–4.01 (m, 2H), 1.29 (dd, *J* = 4.5, 2.0 Hz, 6H) ppm. **^{13}C NMR**: (101 MHz, $CDCl_3$): δ 204.5, 136.3, 133.1, 128.7, 128.6, 43.8, 15.6 ppm.

References

1. Sauthier, M.; Lamotte, N.; Dheur, J.; Castanet, Y.; Mortreux, A., *New J. Chem.* **2009,** *33*, 969-971.
2. Yasuda, M.; Tsuji, S.; Shigeyoshi, Y.; Baba, A., *J. Am. Chem. Soc.* **2002,** *124*, 7440-7447.
3. Mattson, A. E.; Bharadwaj, A. R.; Zuhl, A. M.; Scheidt, K. A., *J. Org. Chem.* **2006,** *71*, 5715-5724.
4. Arceo, E.; Bahamonde, A.; Bergonzini, G.; Melchiorre, P., *Chem. Sci.* **2014,** *5*, 2438-2442.
5. Mu, X. J.; Zou, J., P.; Wanga, Z., T.; Zhang, W., *Tetrahedron Lett.* **2005,** *46*, 4727–4729.
6. Xuan, J.; Feng, Z.-J.; Chen, J.-R.; Lu, L.-Q.; Xiao, W.-J., *Chem. Eur. J.* **2014,** *20*, 3045-3049.
7. Drewes, S. E.; Hogan, C. J.; Kaye, P. T.; Roos, G. H. P., *J. Chem. Soc., Perkin Tran. 1* **1989**, 1585-1591.

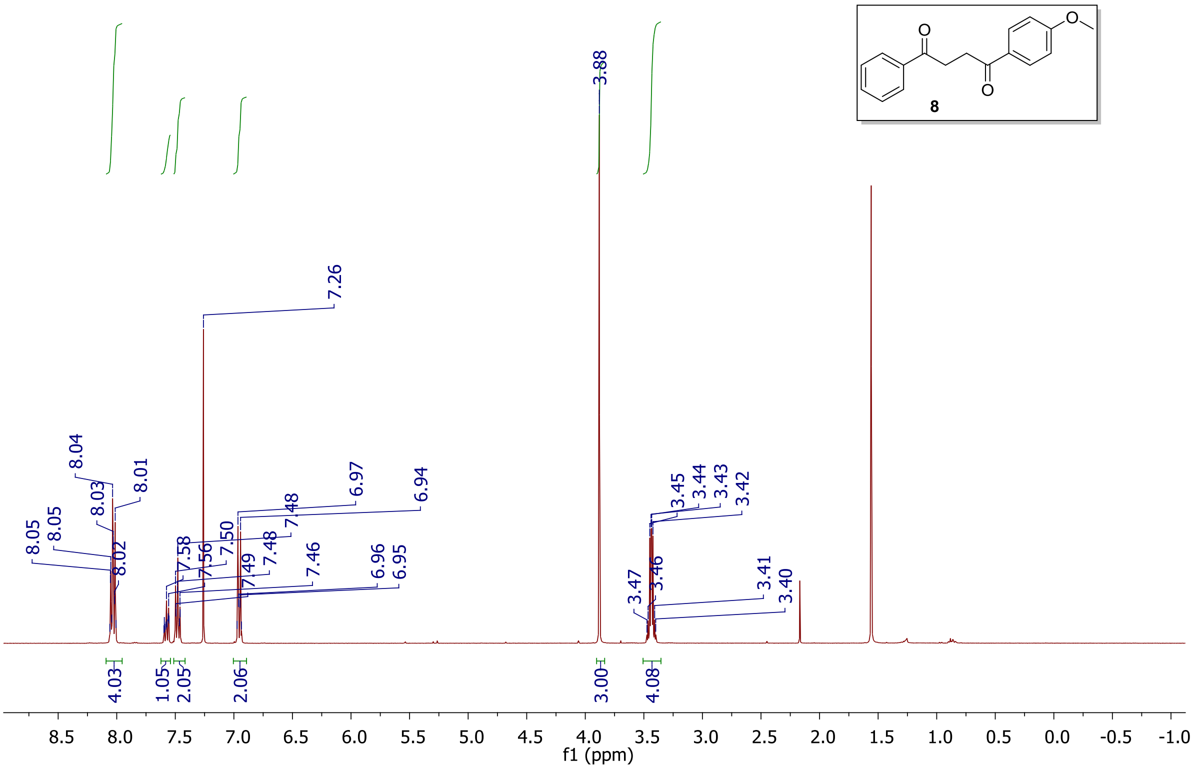

^{1}H NMR of **8** ($CDCl_3$, 400 MHz)

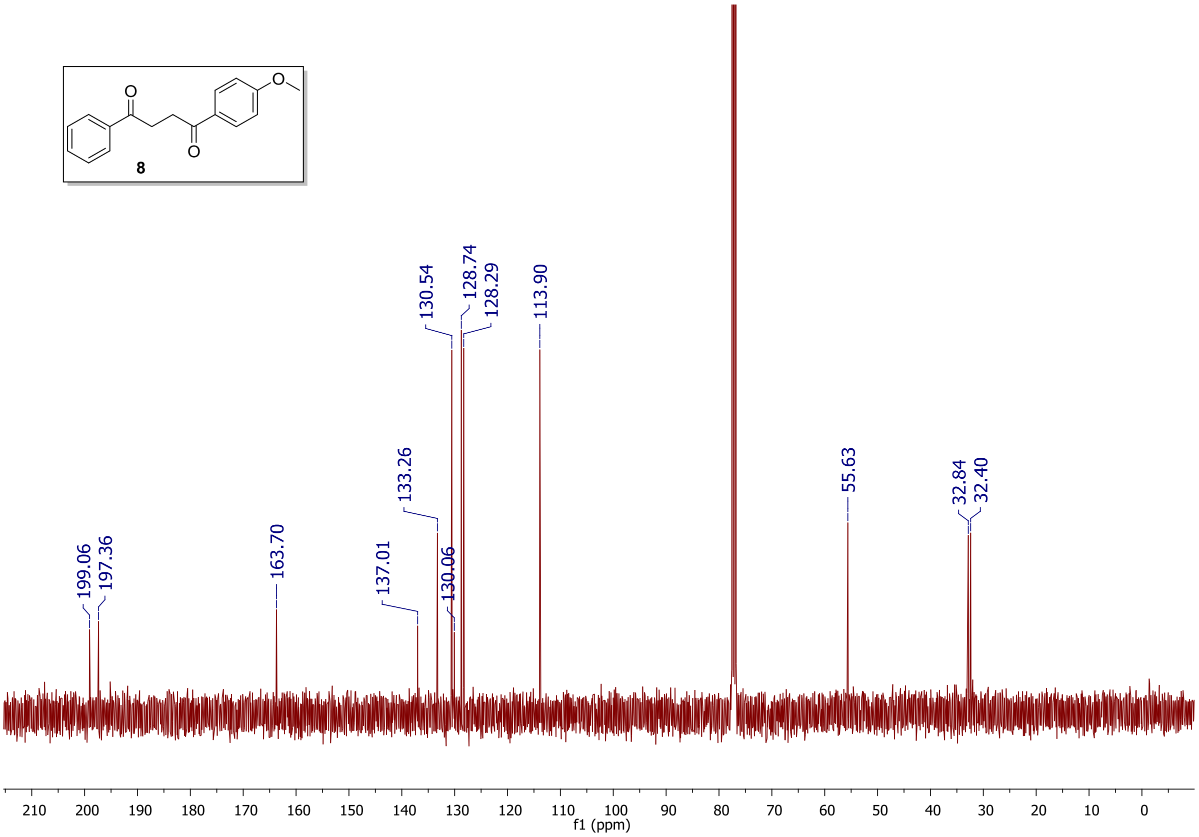

^{13}C NMR of **8** ($CDCl_3$, 101 MHz)

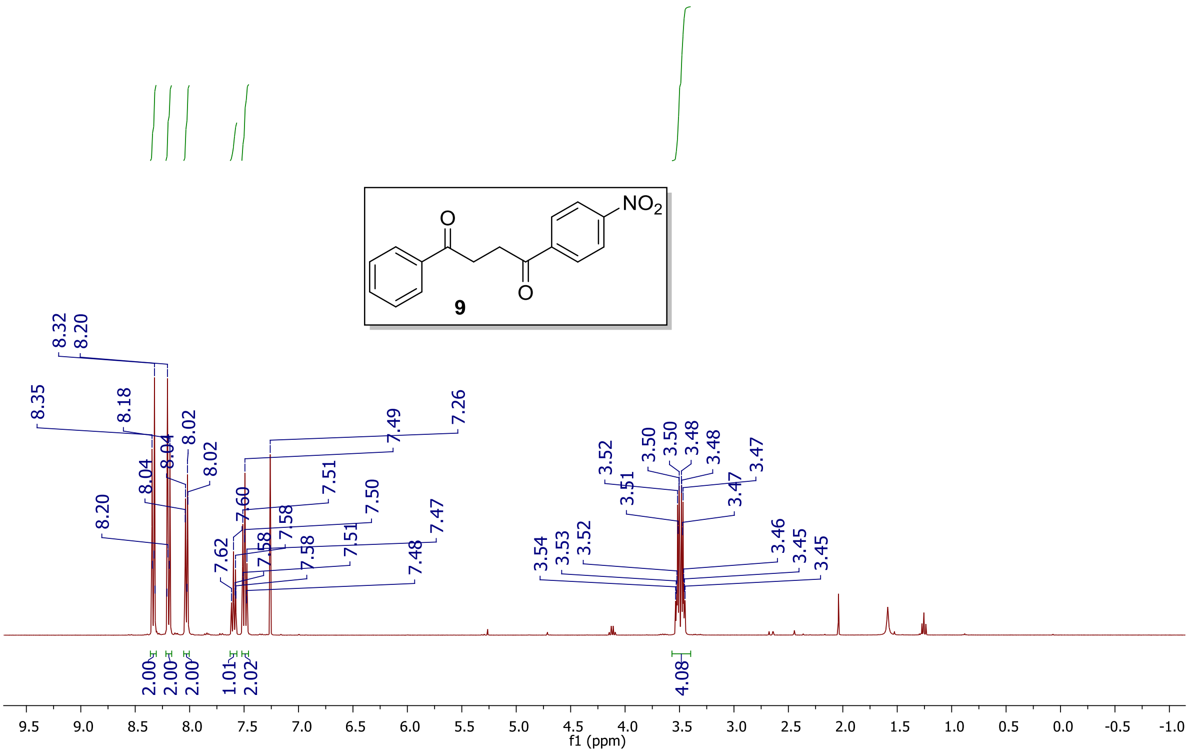

^{1}H NMR of **9** ($CDCl_3$, 400 MHz)

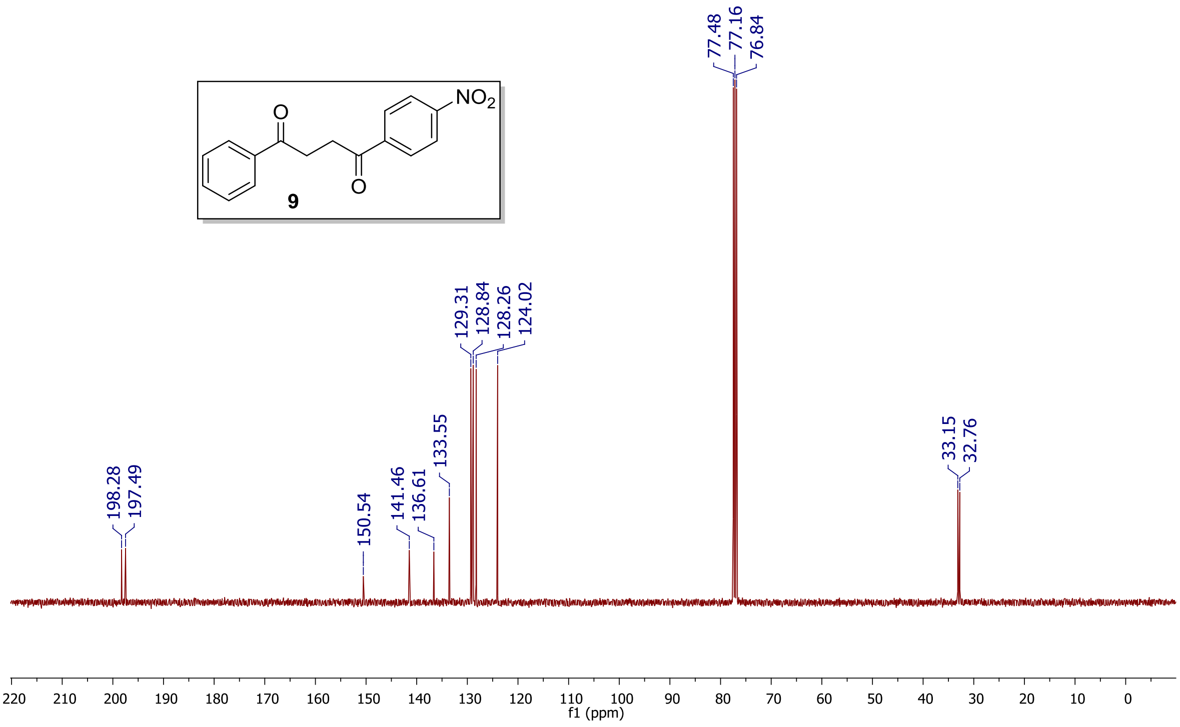

^{13}C NMR of **9** ($CDCl_3$, 101 MHz)

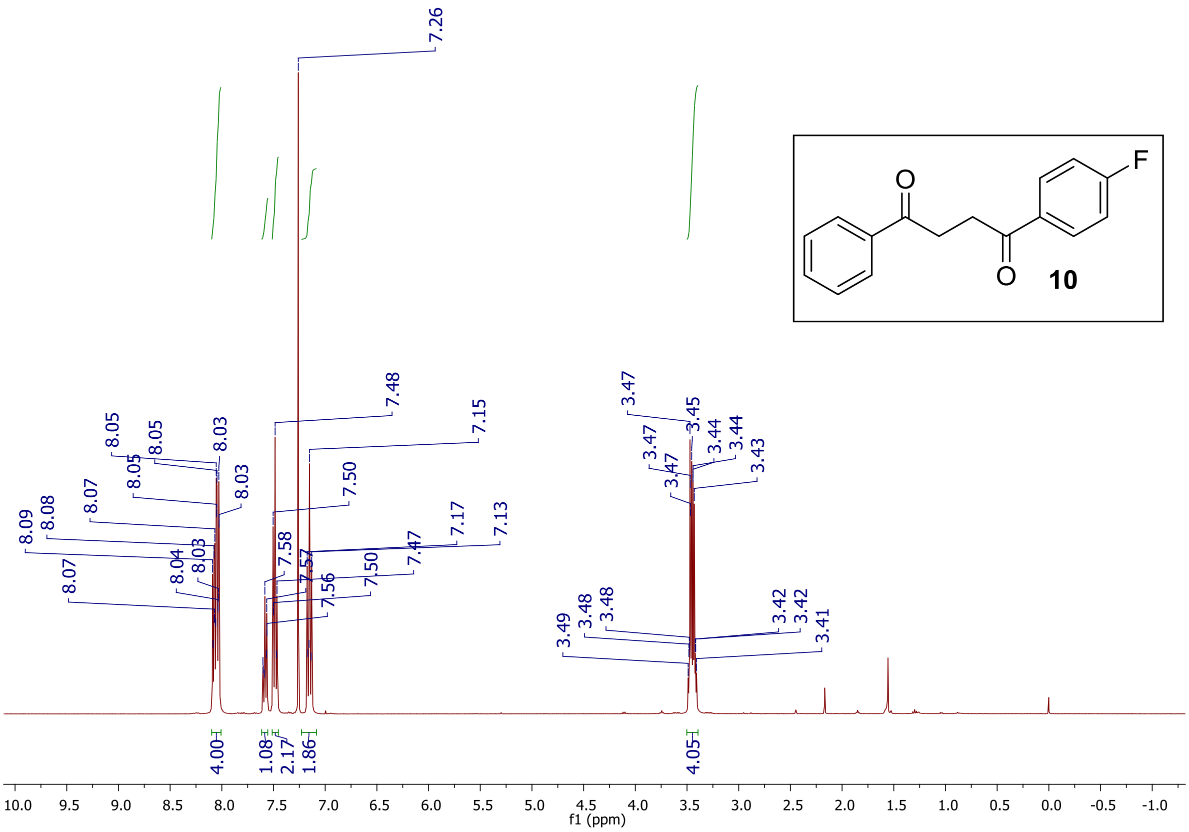

^{1}H NMR of **10** ($CDCl_3$, 400 MHz)

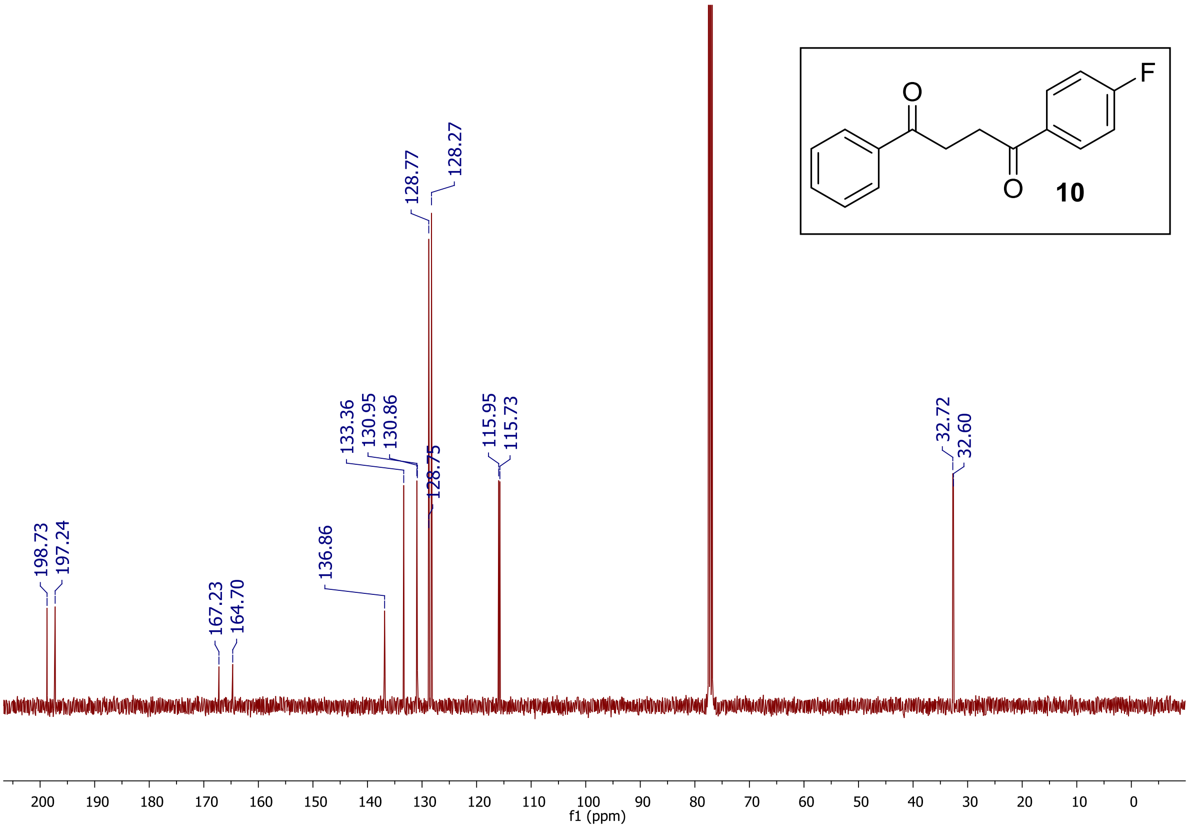

^{13}C NMR of **10** ($CDCl_3$, 101 MHz)

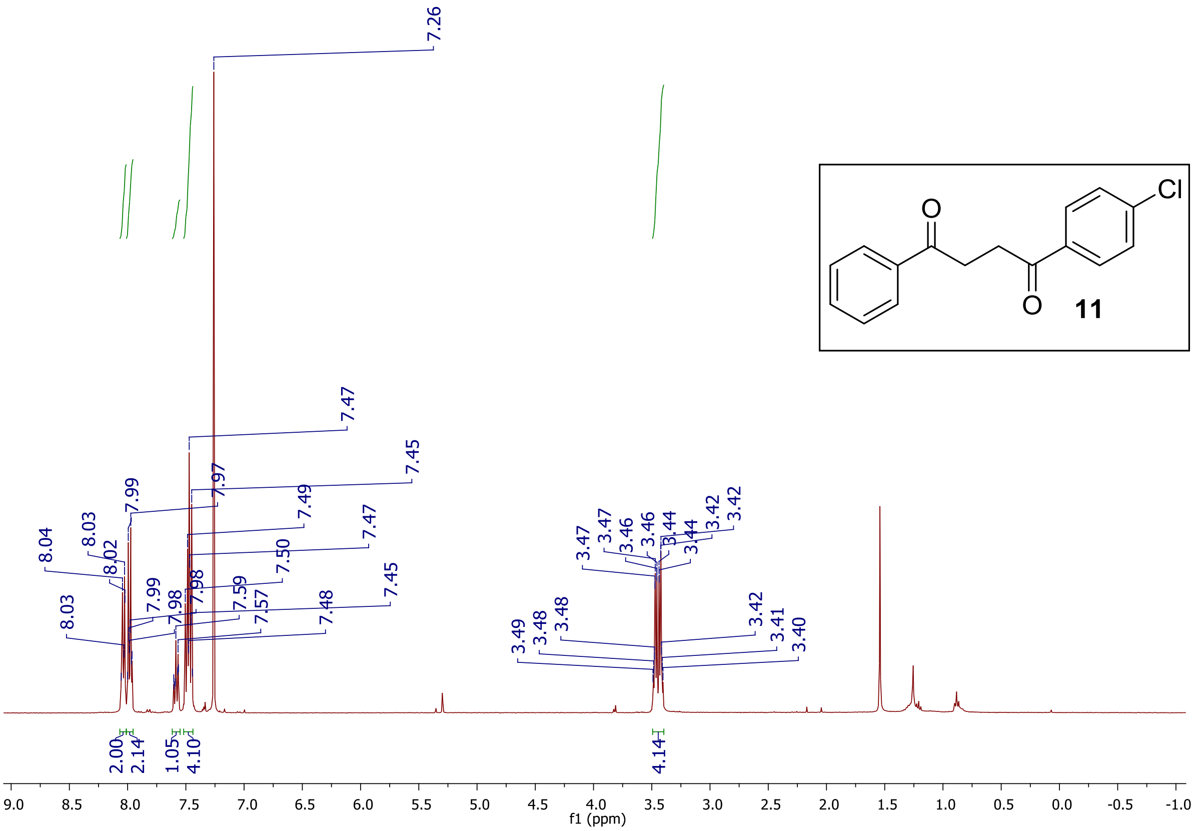

^{1}H NMR of **11** ($CDCl_3$, 400 MHz)

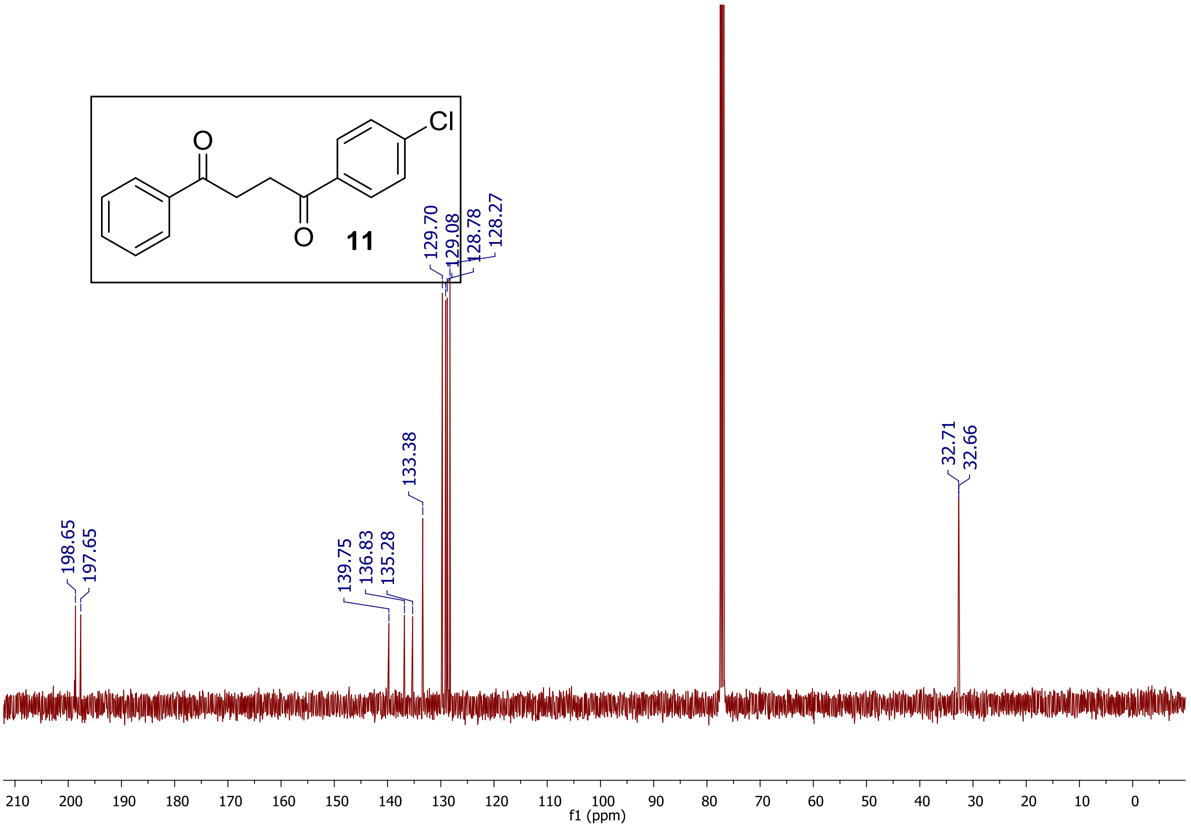

^{13}C NMR of **11** ($CDCl_3$, 101 MHz)

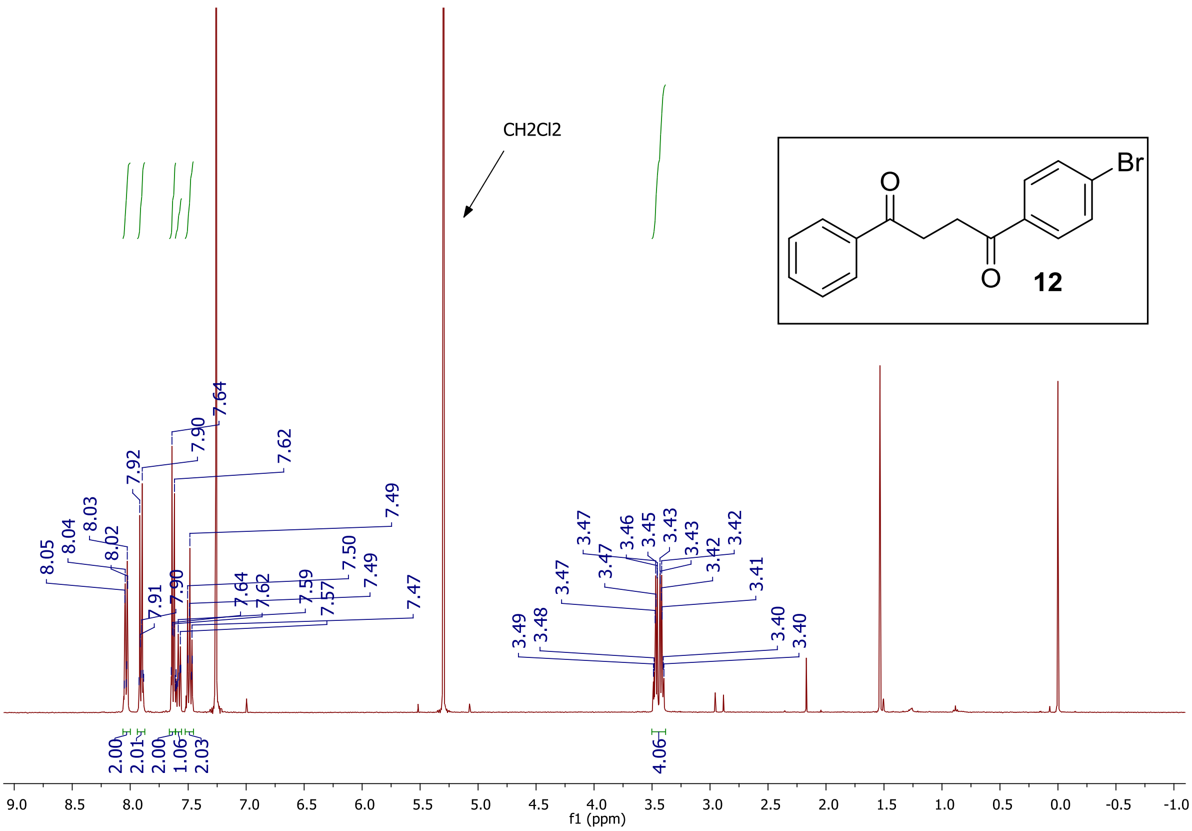

^{1}H NMR of **12** ($CDCl_3$, 400 MHz)

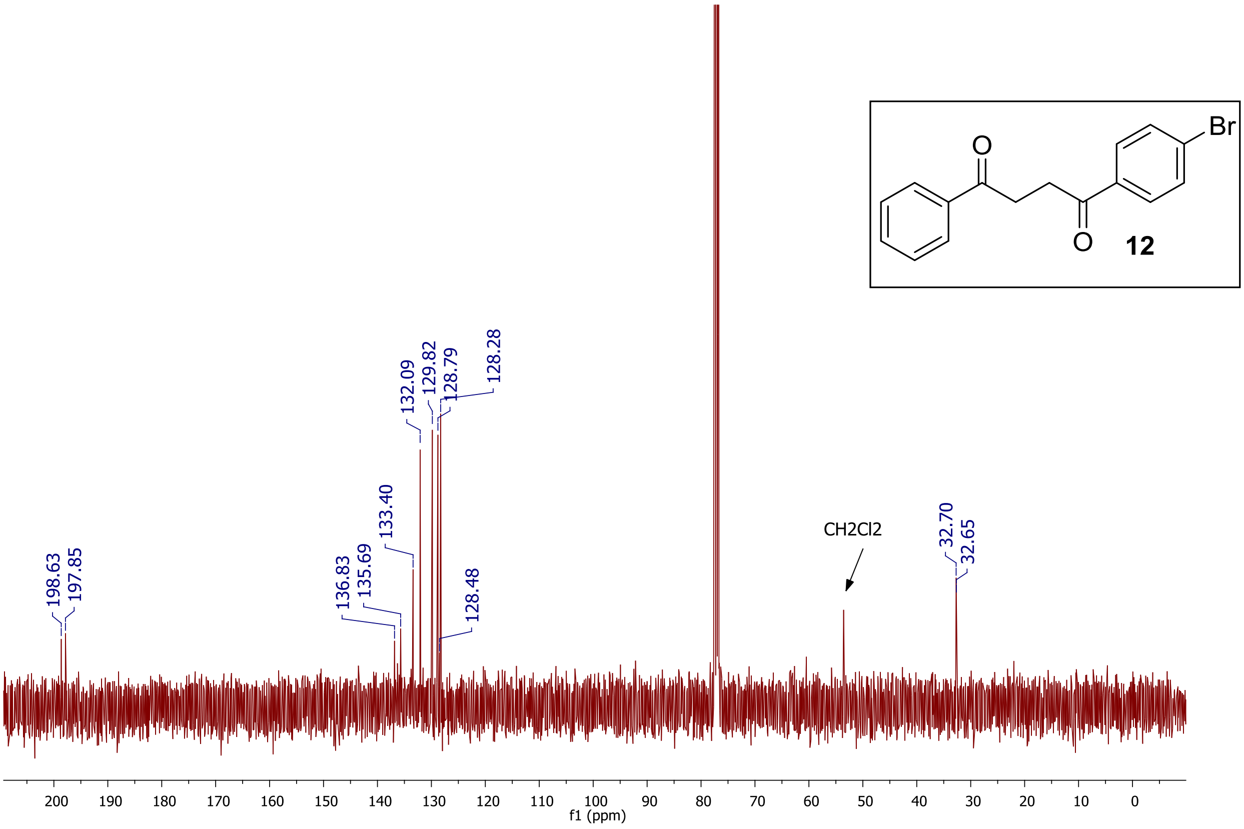

^{13}C NMR of **12** ($CDCl_3$, 101 MHz)

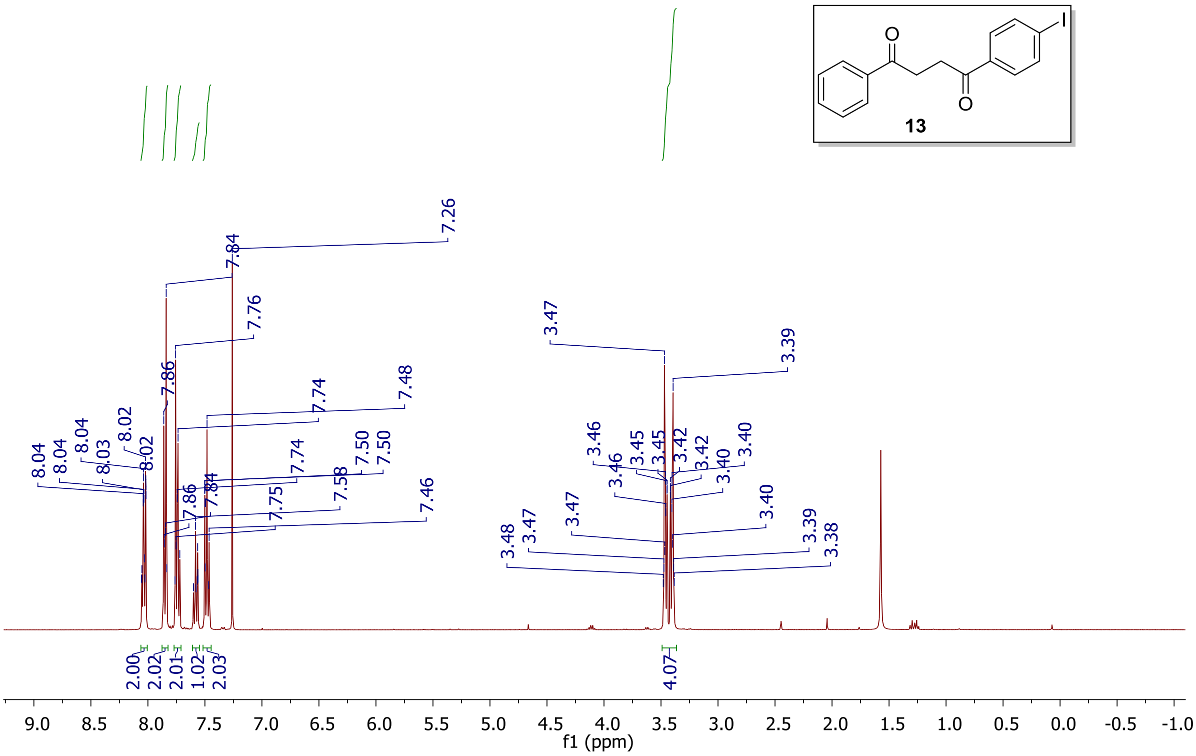

^{1}H NMR of **13** ($CDCl_3$, 400 MHz)

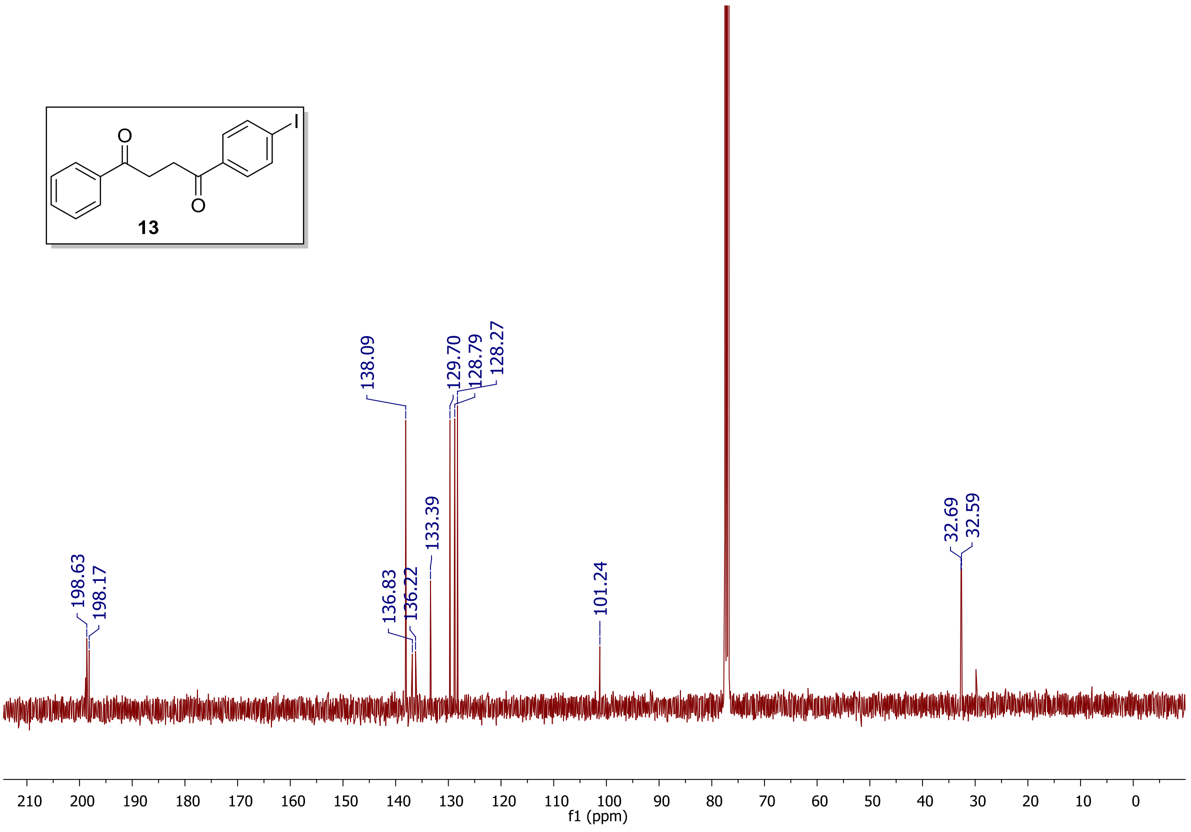

^{13}C NMR of **13** ($CDCl_3$, 101 MHz)

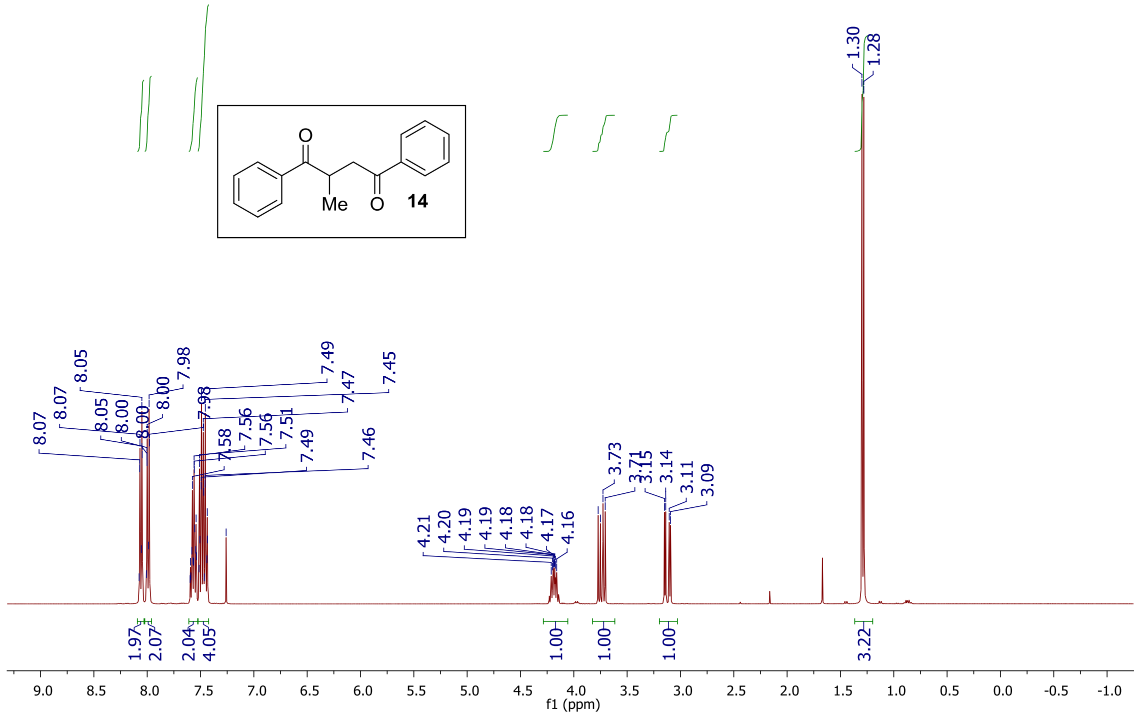

^{1}H NMR of **14** ($CDCl_3$, 400 MHz)

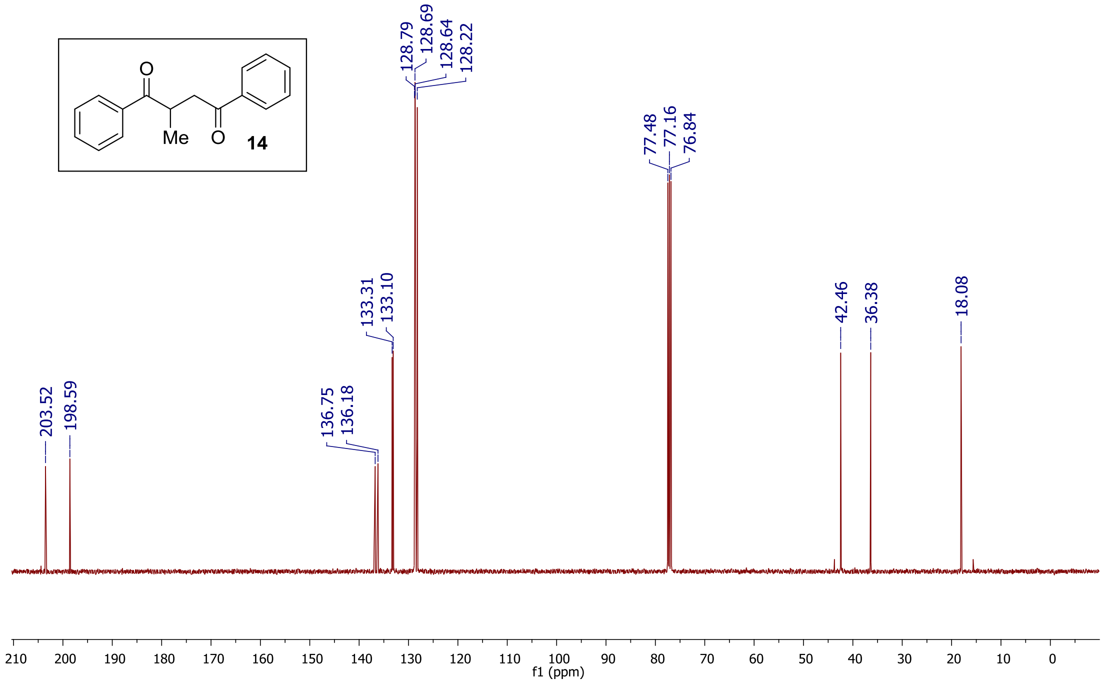

^{13}C NMR of **14** ($CDCl_3$, 101 MHz)

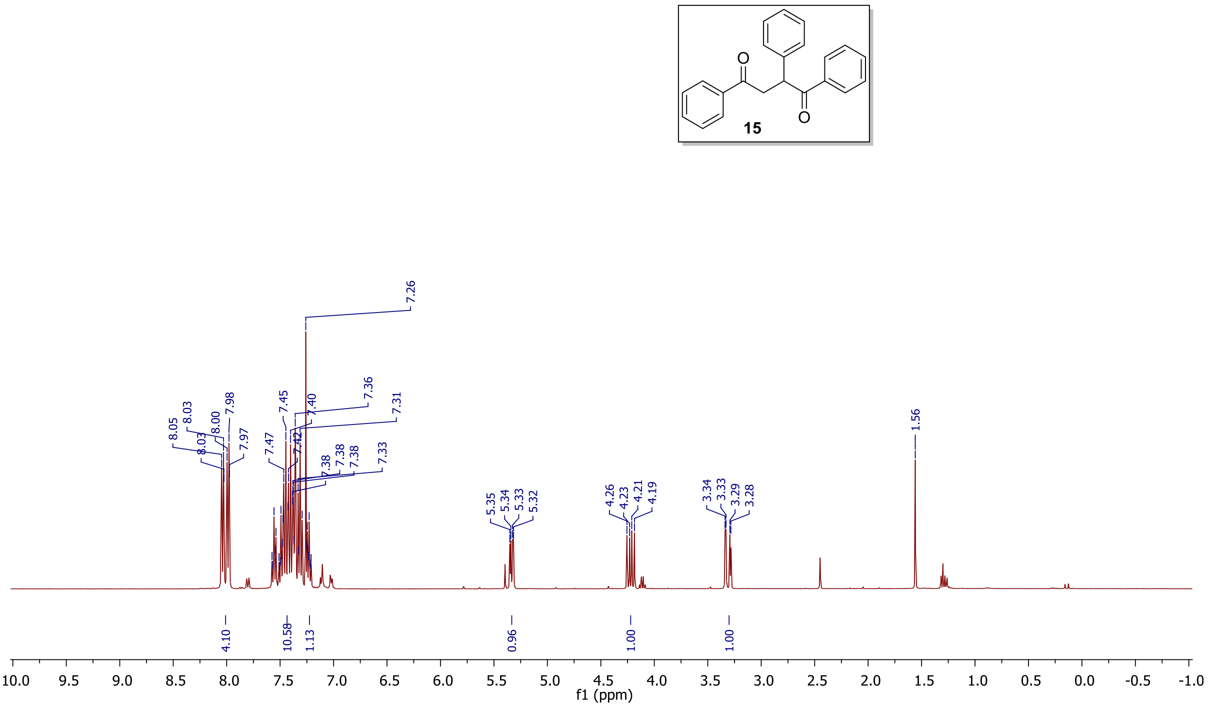

^{1}H NMR of **15** ($CDCl_3$, 400 MHz)

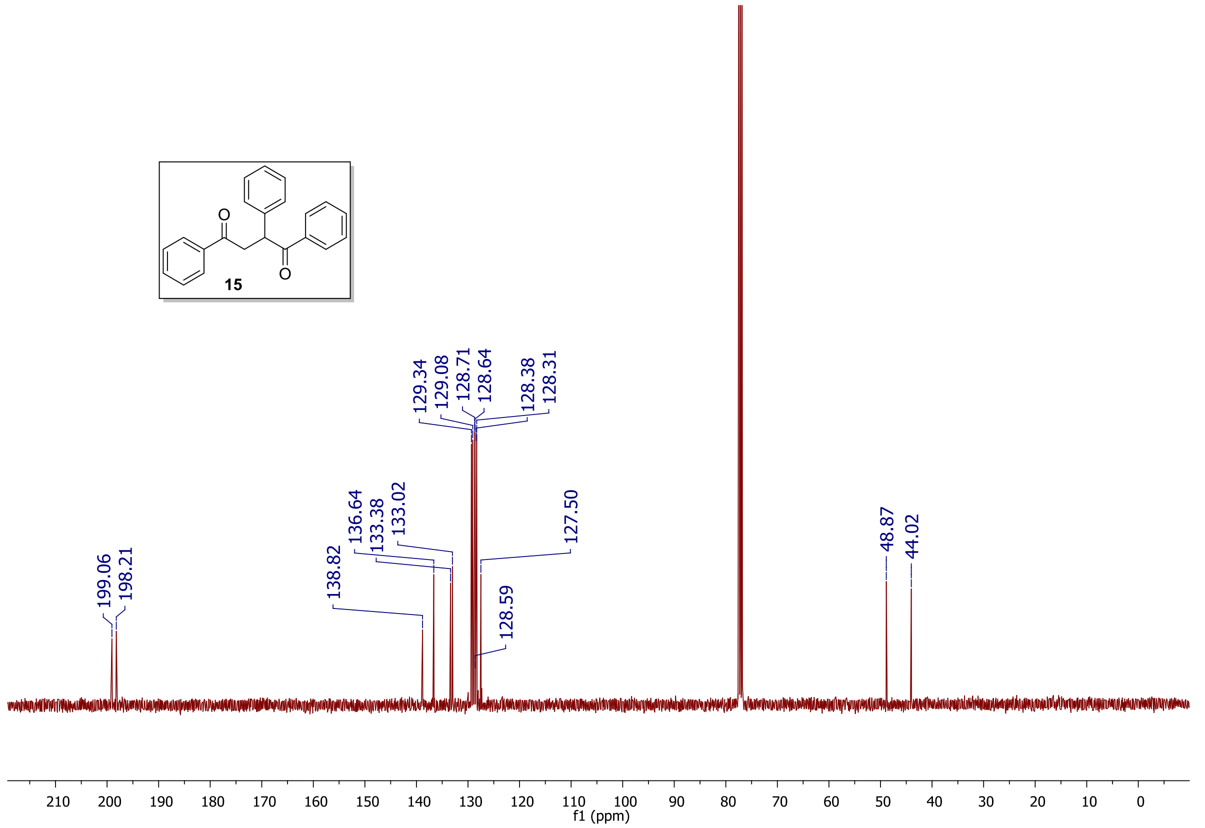

^{13}C NMR of **15** ($CDCl_3$, 101 MHz)

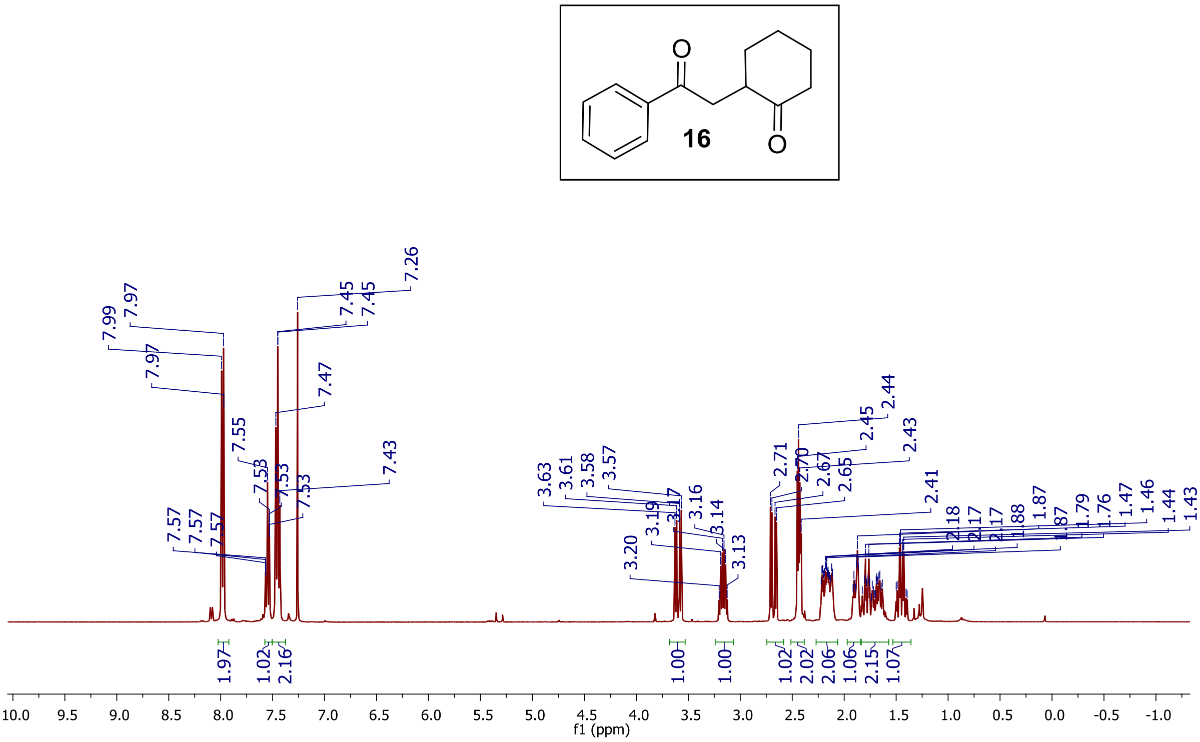

^{1}H NMR of **16** ($CDCl_3$, 400 MHz)

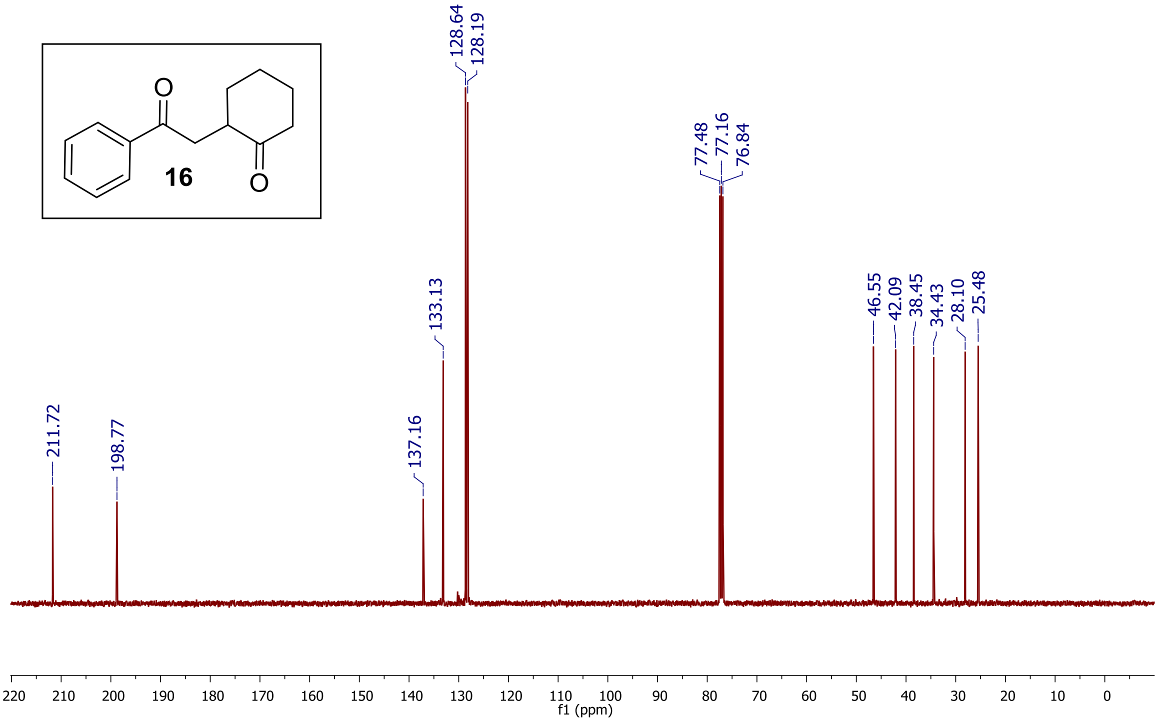

^{13}C NMR of **16** ($CDCl_3$, 101 MHz)

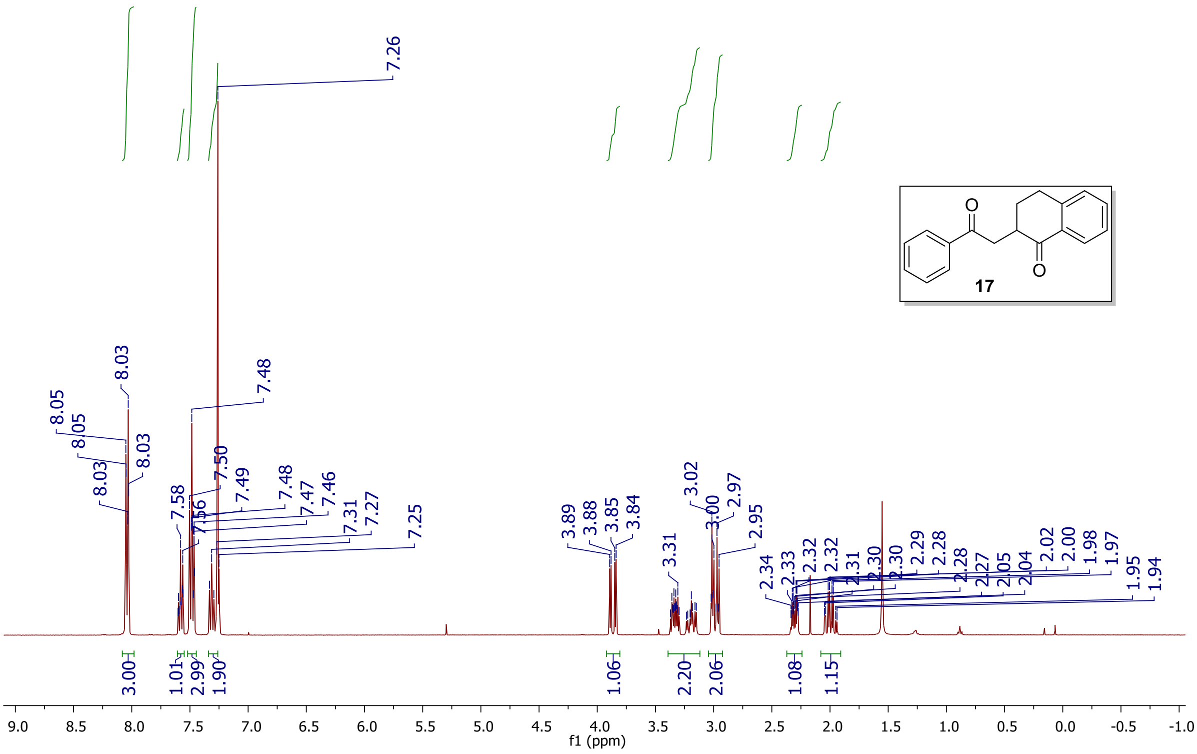

^{1}H NMR of **17** ($CDCl_3$, 400 MHz)

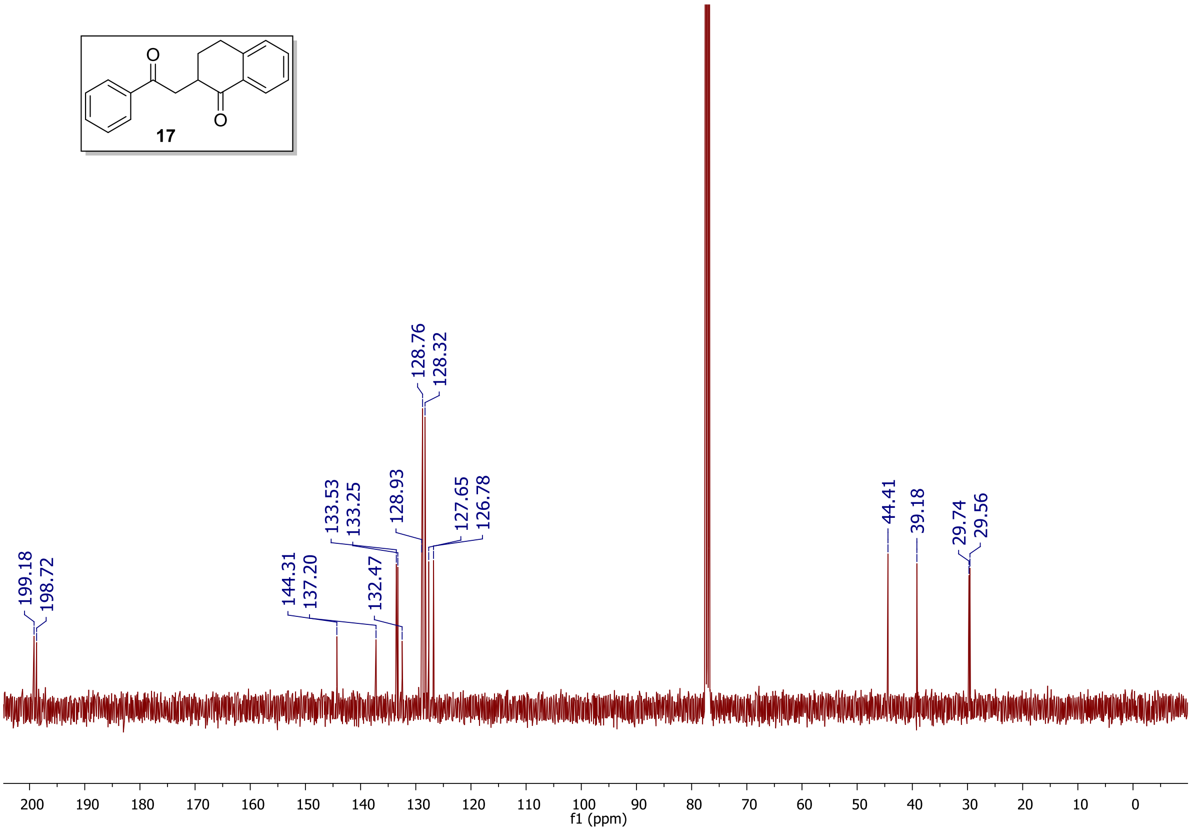

^{13}C NMR of **17** ($CDCl_3$, 101 MHz)

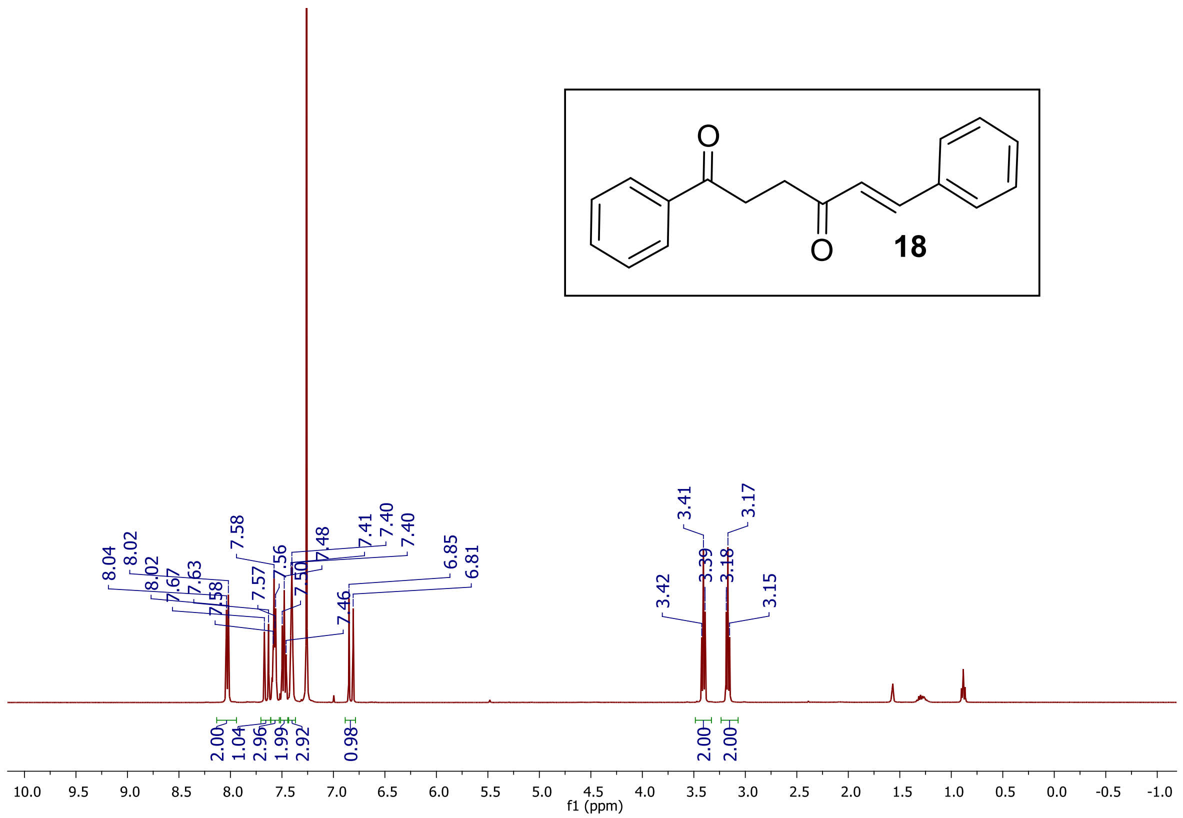

^{1}H NMR of **18** ($CDCl_3$, 400 MHz)

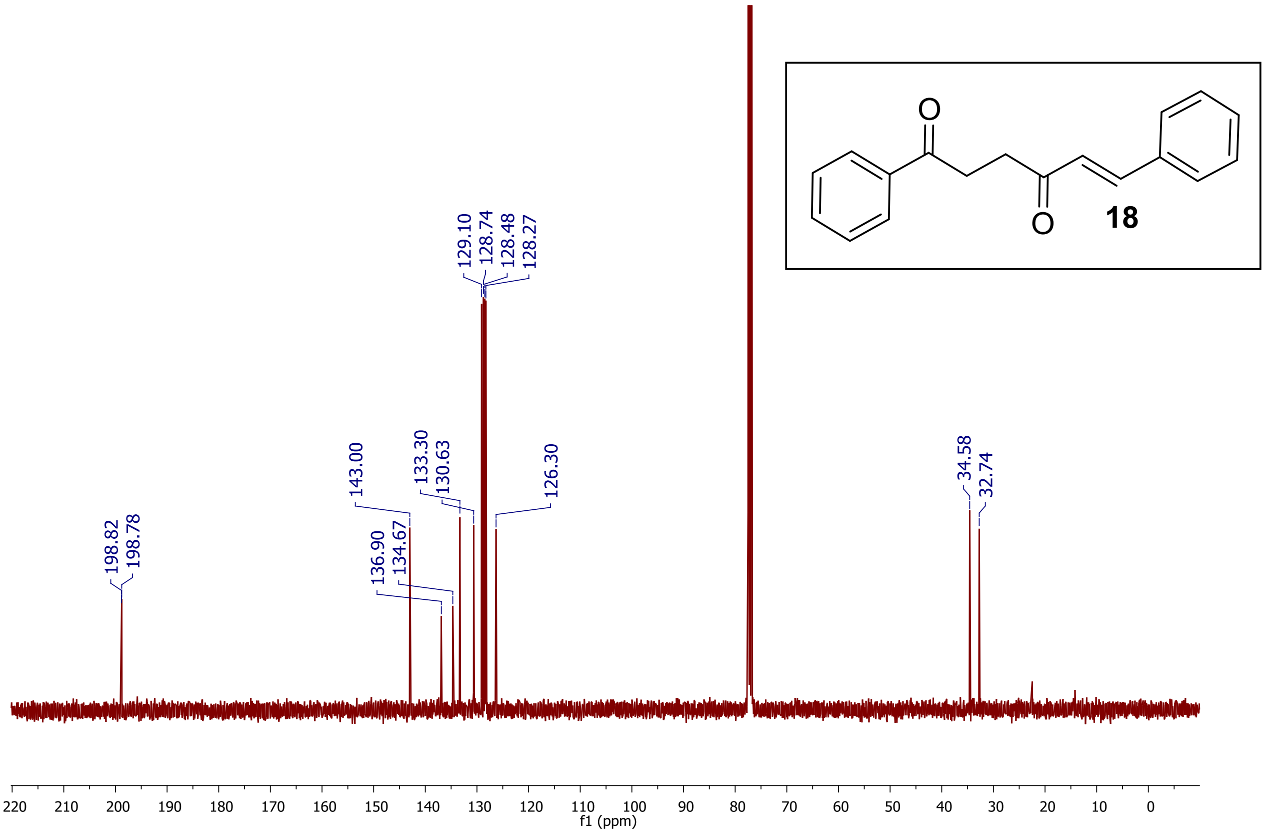

^{13}C NMR of **18** ($CDCl_3$, 101 MHz)

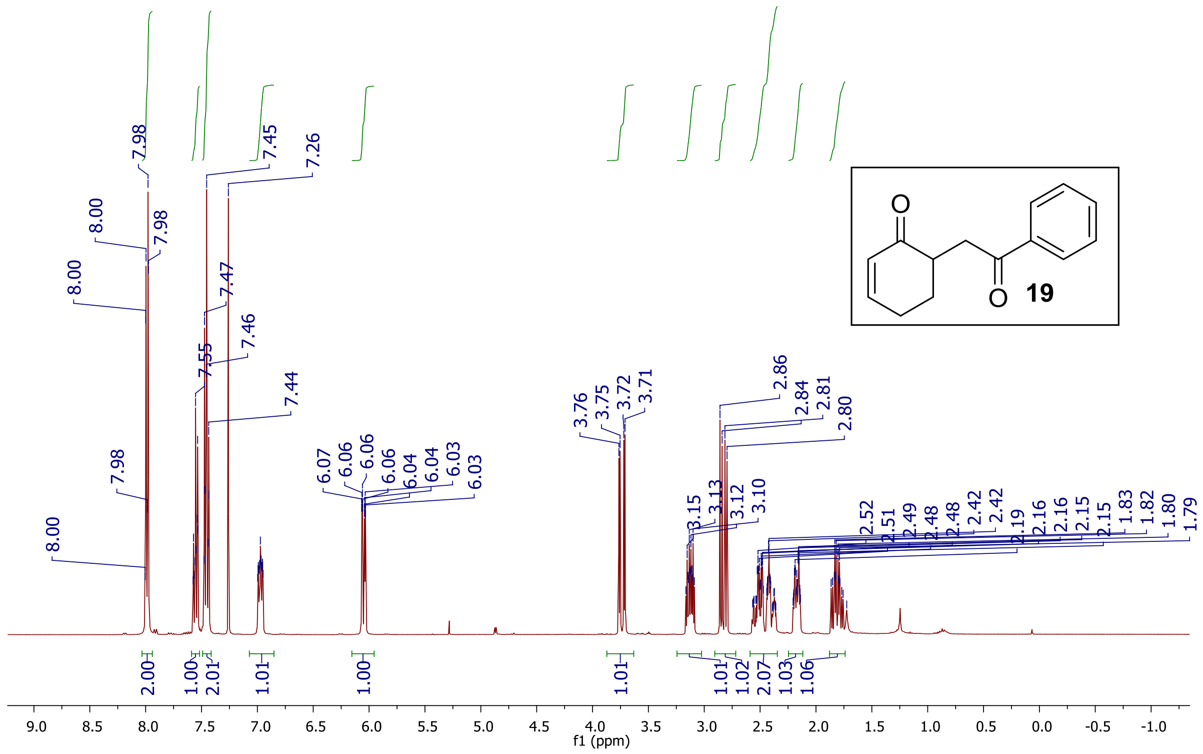

^{1}H NMR of **19** ($CDCl_3$, 400 MHz)

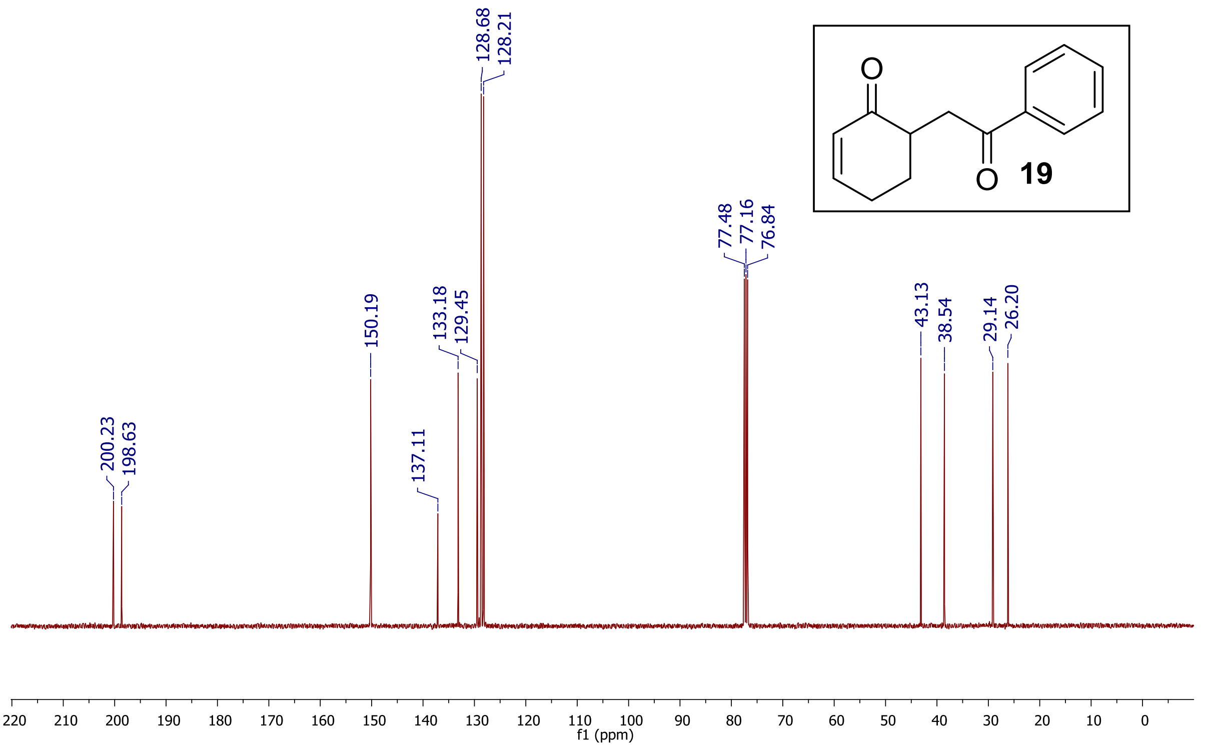

^{13}C NMR of **19** ($CDCl_3$, 101 MHz)

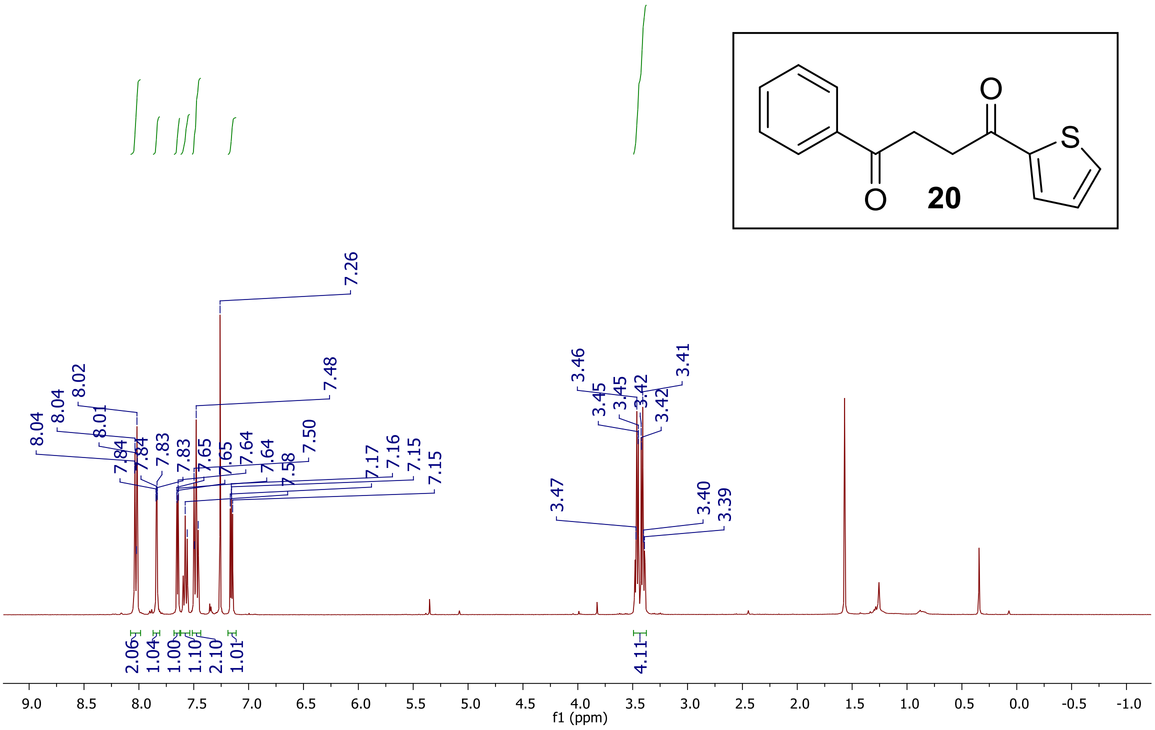

^{1}H NMR of **20** ($CDCl_3$, 400 MHz)

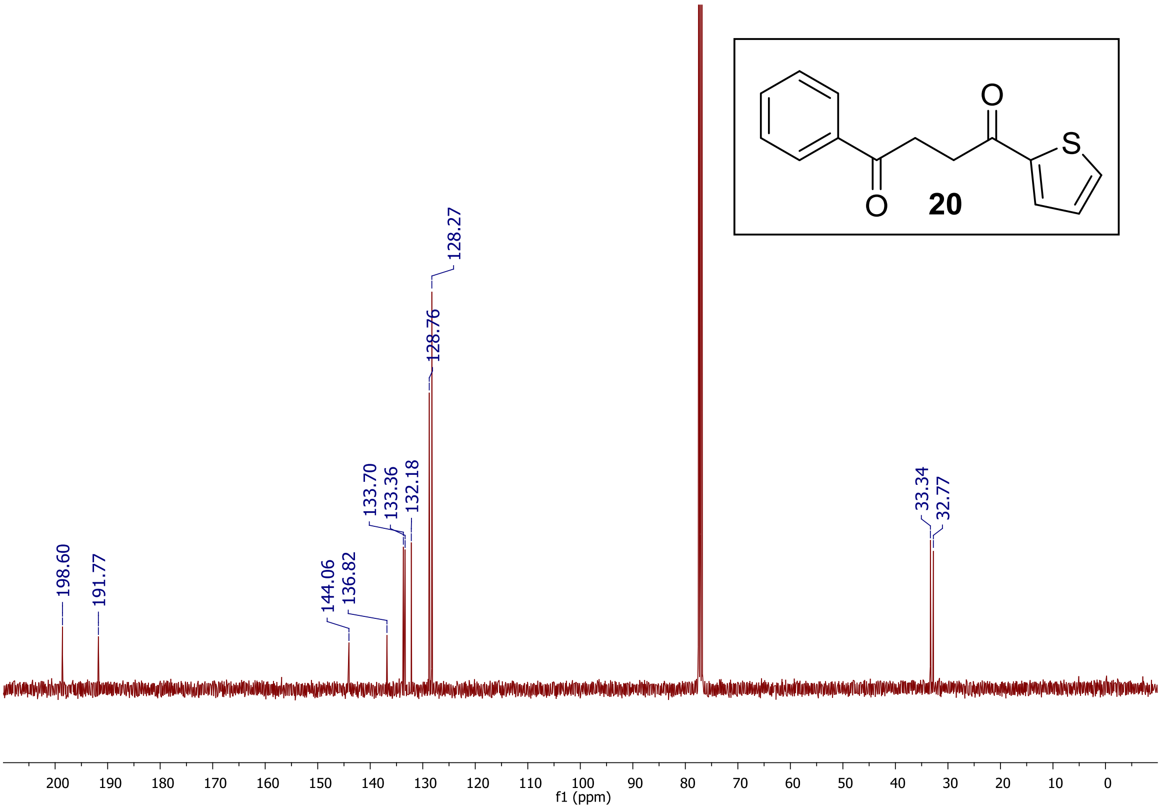

^{13}C NMR of **20** ($CDCl_3$, 101 MHz)

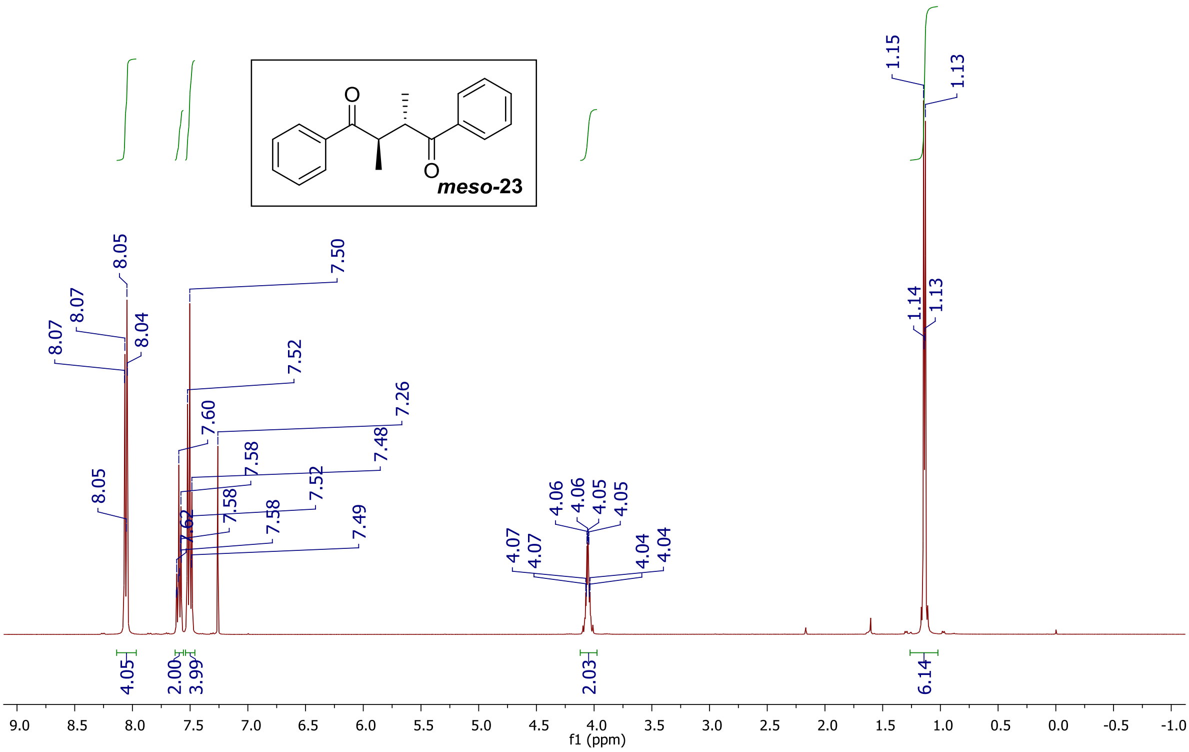

^{1}H NMR of **23** ($CDCl_3$, 400 MHz)

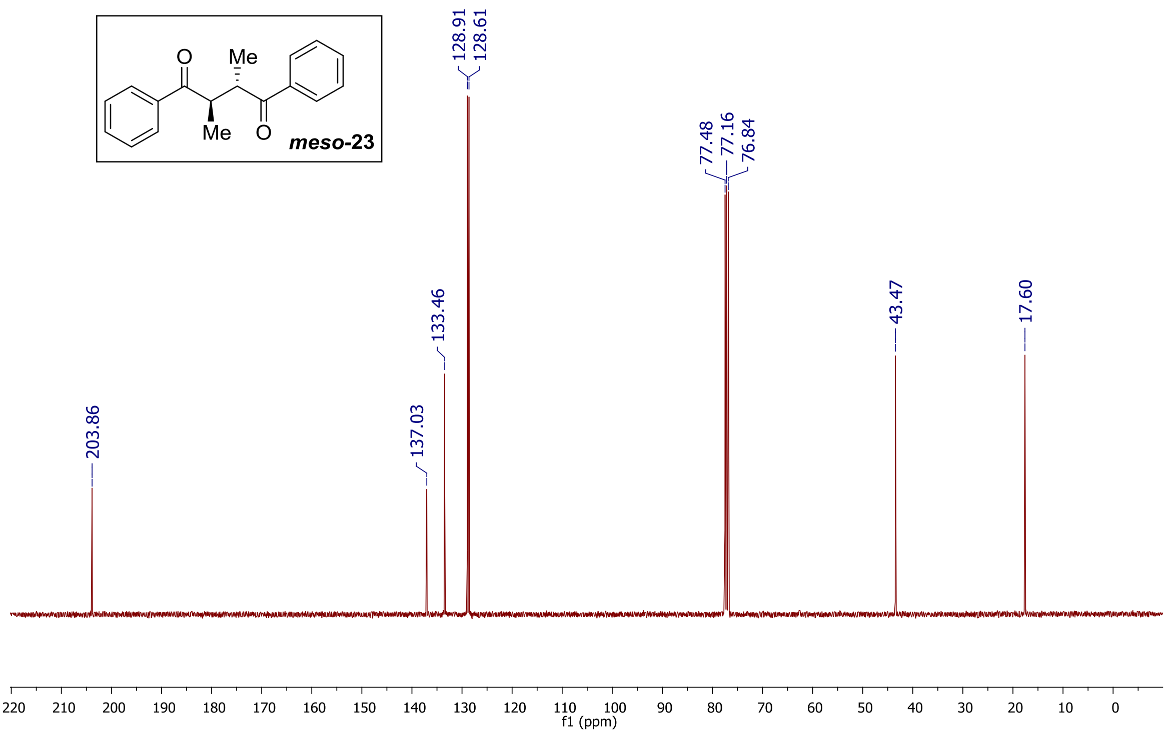

^{13}C NMR of **23** ($CDCl_3$, 101 MHz)

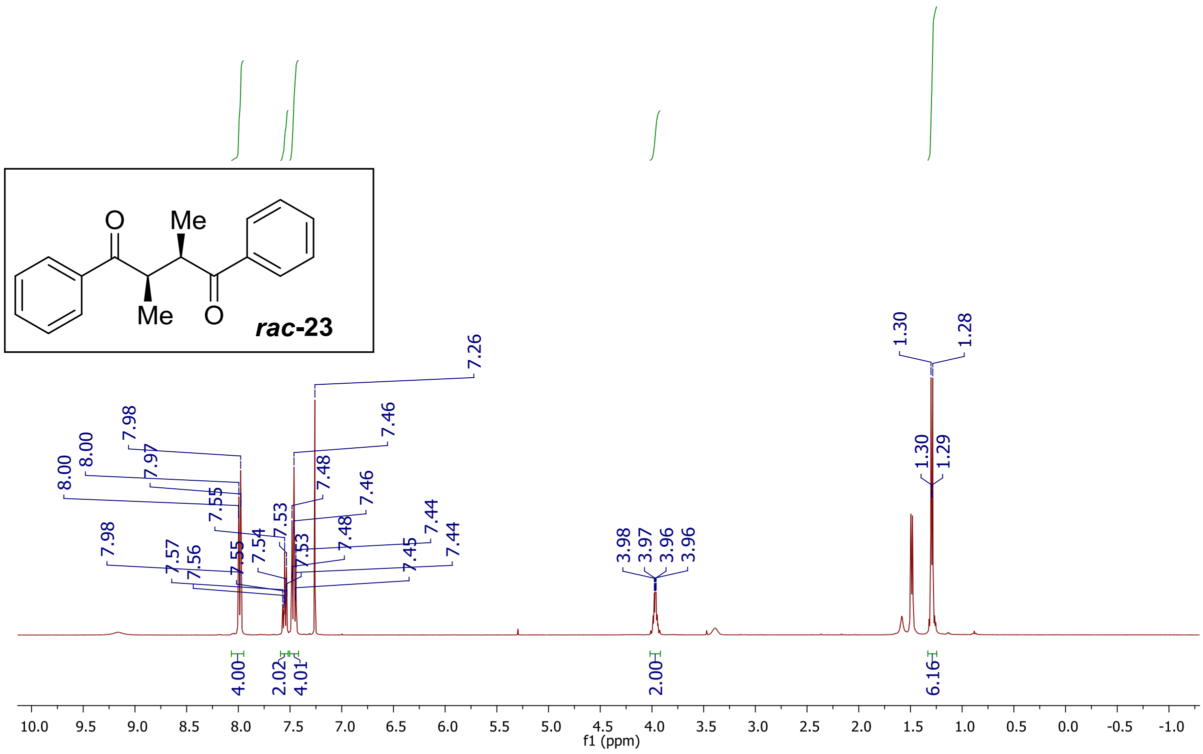

^{1}H NMR of **23** ($CDCl_3$, 400 MHz)

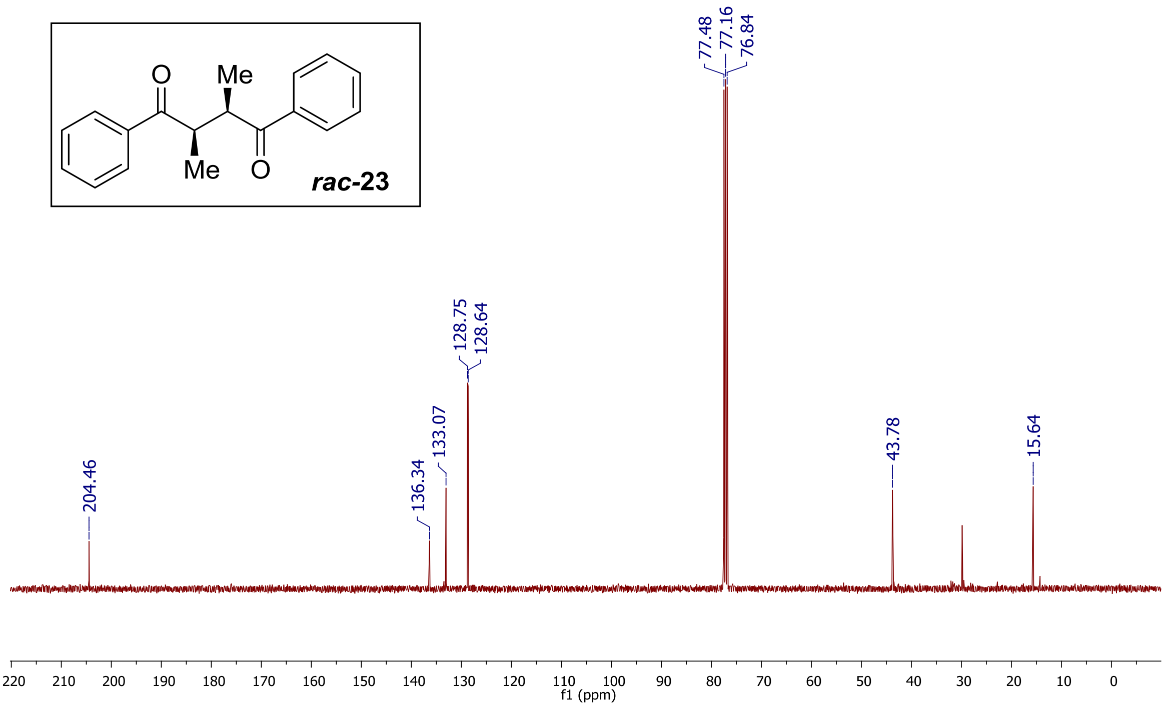

^{13}C NMR of **23** ($CDCl_3$, 101 MHz)
